# Supplementary material for: A methodology and theoretical taxonomy for centrality measures: What are the best centrality indicators for student networks?
Source: PLoS One. 2020 Dec 30;15(12):e0244377. doi: 10.1371/journal.pone.0244377 (PMC7773201; doi:10.1371/journal.pone.0244377)
Supplement: S1 Appendix — (DOCX) [file pone.0244377.s001.docx]

S1 Appendix

Table 1. Studies on networks and centrality, and centrality measures used.

| Field | Reference | Centrality measures used |
| --- | --- | --- |
| Management and organizational contexts | Mehra & al., 2001 | Betweenness centrality |
|  | Sparrowe & al., 2001 | *In*- degree centrality |
|  | Tsai, 2001 | *In*- degree centrality |
|  | Perry-Smith, 2006 | Closeness centrality (Freeman) |
|  | Zhang & Venkatesh, 2013 | *In*- degree centrality  Indirect ties |
|  | Dhand & al., 2014 | Degree centrality  Betweenness centrality  Eigenvector centrality |
|  | Song & al., 2015 | Degree centrality  Betweenness centrality  Flow betweenness centrality  Closeness centrality (Freeman)  Semi-local centrality  Entropy centrality  Residual closeness centrality |
|  | Zedan & Miller, 2017 | Degree centrality  Betweenness centrality  Closeness centrality (Freeman)  Eigenvector centrality  Cross-clique centrality |
| Economy and finance | Montgomery, 1991 | Connectedness |
|  | Calvo-Armengo & Jackson, 2004 | Degree centrality  Indirect ties |
|  | Neal, 2008 | Degree centrality  Betweenness centrality  Closeness centrality (Freeman) |
|  | Clauset & al., 2014 | Closeness centrality (Freeman)  Harmonic centrality |
|  | Ductor & al., 2014 | Degree centrality  Second order centrality degree  Betweenness centrality  Closeness centrality (Freeman)  Giant component |
| Marketing research | Iacobucci & al., 1996 | Degree centrality  Betweenness centrality  Closeness centrality (Freeman) |
|  | Kiss & Bichler, 2008 | Degree centrality  Betweenness centrality  Closeness centrality (Freeman)  Eigenvector centrality  Edge-weighted degree centrality  Kleinberg's authority & hub centralities  Page rank  Weighted Page rank  Sender rank  Weighted sender rank |
|  | Katona & al., 2011 | Degree centrality  Betweenness centrality  Clustering coefficient |
|  | Benoit & Van den Poel, 2012 | Degree centrality  Betweenness centrality |
|  | Kim & al., 2012 | Degree centrality  Degree centralization |
| Field | Reference | Centrality measures used |
| Sociology and political sciences | Krackhardt, 1990 | Degree centrality  Betweenness centrality  Closeness centrality (Freeman) |
|  | Krebs, 2002 | Degree centrality  Betweenness centrality  Closeness centrality (Freeman) |
|  | Chen & al., 2004 | Degree centrality  Betweenness centrality  Closeness centrality (Freeman) |
|  | Burgess & Bruns, 2012 | Degree centrality  Betweenness centrality |
|  | Mastrobuoni & Patacchini, 2012 | Degree centrality  Betweenness centrality  Closeness centrality (Freeman)  Eigenvector centrality  Top rank (citation) |
|  | Faghani & Nguyen, 2013 | Degree centrality  Betweenness centrality  Closeness centrality (Freeman)  Page rank  Cross-clique centrality |
|  | Xu & al., 2014 | Betweenness centrality |
| Biological networks | Idowu & al., 2004 | Hubs  Second order hubs  Bottleneck centrality |
|  | Joy & al., 2005 | Degree centrality  Betweenness centrality |
|  | Yu & al., 2007 | Degree centrality  Betweenness centrality |
|  | Koschützki & Schreiber, 2008 | Degree centrality  Betweenness centrality  Closeness centrality (Freeman)  Radiality centrality  Integration centrality  Katz status index  Page rank  Motif-based centrality |
|  | Lin & al., 2008 | Degree centrality  Edge percolated component  Bottleneck centrality  Maximum neighborhood component  Density of maximum neighborhood component  Subgraph Centrality |
|  | Duran-Pinedo & al., 2011 | Degree centrality  Betweenness centrality  Maximum neighborhood component  Density of maximum neighborhood component |
|  | Wang & al., 2011 | Degree centrality  Betweenness centrality  Closeness centrality (Freeman)  Eigenvector Centrality  Subgraph Centrality  Information Centrality  Edge clustering coefficient |
| Field | Reference | Centrality measures used |
| Biological networks | Doncheva & al., 2012 | Degree centrality  Betweenness centrality  Closeness centrality (Freeman)  Eccentricity centrality  Number of connected components  Average number of neighbors  Neighborhood connectivity of a node  Clustering coefficient  Number of shared neighbors  Topological coefficient  Stress centrality |
|  | Chin & al., 2014 | Degree centrality  Betweenness centrality  Closeness centrality (Freeman)  Eccentricity centrality  Edge percolated component  Maximum neighborhood component  Density of maximum neighborhood component  Maximal clique centrality  Bottleneck centrality  Radiality centrality  Stress centrality |
|  | Peng & al., 2015 | Localization-specific centrality score combined with 7 centrality measures :  Degree centrality  Betweenness centrality  Closeness centrality (Freeman)  Eigenvector Centrality  Subgraph Centrality  Information Centrality  Edge clustering coefficient |
|  | Ashtiani & al., 2018 | Degree centrality  Betweenness centrality  Closeness centrality (Freeman)  Closeness centrality (Latora)  Average Distance  Barycenter centrality  Residual closeness centrality  ClusterRank  Decay centrality  Diffusion degree  Maximum neighborhood component  Density of maximum neighborhood component  Geodesic *k*-path centrality  Katz status index  Laplacian centrality  Leverage centrality  Lin centrality  Lobby index (centrality)  Markov centrality  Radiality centrality  Eigenvector centrality  Subgraph Centrality  Eccentricity centrality  Kleinberg's authority & hub centralities  Harary centrality  Information centrality |
| Field | Reference | Centrality measures used |
| Student networks : performance, achievement and learning | Thomas, 2000 | Degree centrality  Bonacich centrality |
|  | Yang and Tang, 2003 | *In*- degree centrality |
|  | Russo & Koesten, 2005 | Degree centrality |
|  | Cho & al., 2007 | Degree centrality  Closeness centrality (Freeman) |
|  | De Laat & al., 2007 | Degree centrality |
|  | Zhang & al., 2008 | Closeness centrality (Freeman) |
|  | Obadi & al., 2010 | Degree centrality  Betweenness centrality  Closeness centrality (Freeman) |
|  | Hommes & al., 2012 | Degree centrality  Betweenness centrality |
|  | Woolf & al., 2012 | Closeness centrality (Freeman) |
|  | Bruun & Brewe, 2013 | *In*- degree centrality  *In*- strength centrality  Page rank  Hide centrality  Target entropy |
|  | Gašević & al., 2013 | Degree centrality  Betweenness centrality  Closeness centrality (Freeman)  Eccentricity centrality |
|  | Vaughan & al., 2015 | Degree centrality |
|  | de-Marcos & al., 2016 | Degree centrality  Betweenness centrality  Closeness centrality (Freeman)  Eccentricity centrality  Eigenvector centrality  Kleinberg's authority & hub centralities  Page rank  Clustering coefficient |
|  | Mushtaq & al., 2016 | Degree centrality  Betweenness centrality  Closeness centrality (Freeman) |
|  | Poldin & al., 2016 | Degree centrality |
|  | Zwolak & al., 2017 | *Out*-degree centrality  Betweenness centrality  Closeness centrality (Freeman)  Eigenvector centrality |
|  | Liu & al., 2018 | Degree centrality  Betweenness centrality  Closeness centrality (Freeman) |
|  | Saqr & al., 2018a | Degree centrality  Betweenness centrality  Closeness centrality (Freeman)  Eigenvector centrality  Eccentricity centrality  Information centrality  Clustering coefficient |
|  | Saqr & al., 2018b | Degree centrality  Betweenness centrality  Closeness centrality (Freeman)  Eigenvector centrality  Eccentricity centrality  Information centrality  Clustering coefficient  Indirect ties |
| Field | Reference | Centrality measures used |
| Student networks : performance, achievement and learning | Vargas & al., 2018 | Degree centrality  Betweenness centrality  Closeness centrality (Freeman)  Net out-strength  *Out*- & *in*- disparities  Local clustering  Harmonic centrality |
|  | Vignery & Laurier, 2020 | Degree centrality |
| Student networks : delinquency | Baerveldt & al., 2004 | Degree centrality  Connectedness |
|  | Cruz & al., 2012 | Closeness centrality (Freeman) |
| Student networks : sense of community | Ennett & al., 2006 | *In*- degree centrality  Betweenness centrality  Reach centrality  Bonacich centrality |
|  | Dawson, 2008 | Degree centrality  Betweenness centrality  Closeness centrality (Freeman) |
| Student networks : dropout | Bayer & al., 2012 | Degree centrality  Betweenness centrality |
|  | Yang & al., 2013 | Degree centrality  Betweenness centrality  Closeness centrality (Freeman)  Eigenvector centrality  Average clustering coefficient  Eccentricity centrality  Kleinberg's authority & hub centralities |

Table 2. Centrality indices used in all 63 studies on networks: number of uses and percentages

| Indices  (Total = 248 indices used in the 63 studies) | Number of uses  (among the 63 studies) | % of uses  (among the 63 studies) |
| --- | --- | --- |
| Degree centrality (*in*- and/or *out*-) | 54 | 85.71% |
| Betweenness centrality (Freeman) | 40 | 63.49% |
| Closeness centrality (Freeman) | 34 | 53.97% |
| Eigenvector centrality | 12 | 19.05% |
| Eccentricity centrality | 8 | 12.70% |
| Clustering coefficient | 5 | 7.94% |
| Information Centrality | 5 | 7.94% |
| Page rank | 5 | 7.94% |
| Density of maximum neighborhood component | 4 | 6.35% |
| Kleinberg's authority & hub centralities | 4 | 6.35% |
| Maximum neighborhood component | 4 | 6.35% |
| Subgraph Centrality | 4 | 6.35% |
| Bottleneck centrality | 3 | 4.76% |
| Indirect ties | 3 | 4.76% |
| Radiality centrality | 3 | 4.76% |
| Bonacich centrality | 2 | 3.17% |
| Connectedness | 2 | 3.17% |
| Cross-clique centrality | 2 | 3.17% |
| Edge clustering coefficient | 2 | 3.17% |
| Edge percolated component | 2 | 3.17% |
| Harmonic centrality | 2 | 3.17% |
| Katz status index | 2 | 3.17% |
| Residual closeness centrality | 2 | 3.17% |
| Stress centrality | 2 | 3.17% |
| Average clustering coefficient | 1 | 1.59% |
| Average Distance | 1 | 1.59% |
| Average number of neighbors | 1 | 1.59% |
| Barycenter centrality | 1 | 1.59% |
| Closeness centrality (Latora) | 1 | 1.59% |
| ClusterRank | 1 | 1.59% |
| Decay centrality | 1 | 1.59% |
| Degree centralization | 1 | 1.59% |
| Diffusion degree | 1 | 1.59% |
| Edge-weighted degree centrality | 1 | 1.59% |
| Entropy centrality | 1 | 1.59% |
| Flow betweenness centrality | 1 | 1.59% |
| Geodesic *k*-path centrality | 1 | 1.59% |
| Giant component | 1 | 1.59% |
| Harary centrality | 1 | 1.59% |
| Hide centrality | 1 | 1.59% |
| Hubs | 1 | 1.59% |
| *In*- strength centrality | 1 | 1.59% |
| Integration centrality | 1 | 1.59% |
| Laplacian centrality | 1 | 1.59% |
| Leverage centrality | 1 | 1.59% |
| Lin centrality | 1 | 1.59% |
| Lobby index (centrality) | 1 | 1.59% |
| Local clustering | 1 | 1.59% |
| Markov centrality | 1 | 1.59% |
| Maximal clique centrality | 1 | 1.59% |
| Motif-based centrality | 1 | 1.59% |
| Neighborhood connectivity of a node | 1 | 1.59% |
| Net *out*-strength | 1 | 1.59% |
| Number of connected components | 1 | 1.59% |
| Number of shared neighbors | 1 | 1.59% |
| *Out*- & *in*- disparities | 1 | 1.59% |
| Indices  (Total = 248 indices used in the 63 studies) | Number of uses  (among the 63 studies) | % of uses  (among the 63 studies) |
| Reach centrality | 1 | 1.59% |
| Second order centrality degree | 1 | 1.59% |
| Second order hubs | 1 | 1.59% |
| Semi-local centrality | 1 | 1.59% |
| Sender rank | 1 | 1.59% |
| Target entropy | 1 | 1.59% |
| Top rank (citation) | 1 | 1.59% |
| Topological coefficient | 1 | 1.59% |
| Weighted Page rank | 1 | 1.59% |
| Weighted sender rank | 1 | 1.59% |

Table 3. Centrality indices used in the 27 studies on student networks: number of uses and percentages

| Indices  (Total = 85 indices used in the 27 studies) | Number of uses  (among the 27 studies) | % of uses  (among the 27 studies) |
| --- | --- | --- |
| Degree centrality (*in*- and/or *out*-) | 24 | 88.89% |
| Closeness centrality (Freeman) | 15 | 55.56% |
| Betweenness centrality (Freeman) | 14 | 51.85% |
| Eccentricity centrality | 5 | 18.52% |
| Eigenvector centrality | 5 | 18.52% |
| Clustering coefficient | 3 | 11.11% |
| Bonacich centrality | 2 | 7.41% |
| Information centrality | 2 | 7.41% |
| Kleinberg's authority & hub centralities | 2 | 7.41% |
| Page rank | 2 | 7.41% |
| Average clustering coefficient | 1 | 3.70% |
| Connectedness | 1 | 3.70% |
| Harmonic centrality | 1 | 3.70% |
| Hide centrality | 1 | 3.70% |
| *In*- strength centrality | 1 | 3.70% |
| Indirect ties | 1 | 3.70% |
| Local clustering | 1 | 3.70% |
| Net out-strength | 1 | 3.70% |
| *Out*- & *in*- disparities | 1 | 3.70% |
| Reach centrality | 1 | 3.70% |
| Target entropy | 1 | 3.70% |
