# Supplementary material for: A methodology and theoretical taxonomy for centrality measures: What are the best centrality indicators for student networks?
Source: PLoS One. 2020 Dec 30;15(12):e0244377. doi: 10.1371/journal.pone.0244377 (PMC7773201; doi:10.1371/journal.pone.0244377)
Supplement: S2 Appendix — (DOCX) [file pone.0244377.s002.docx]

S2 Appendix. College student networks' : some studies related to the links between centrality and academic achievement.

| **References** | **Statistical Tools** | **Perspective** | **N Valid** | **Dependent Variable(s)** | **Independent Variables(s)** | **Nature of Impact(s)**  **or of link(s)** |
| --- | --- | --- | --- | --- | --- | --- |
| Thomas (2000) | Hierarchical clustering algorithm and structural equation models | Longitudinal | 322 | GPA | Centrality *out*-degree | Significantly negative |
|  |  |  |  |  | Centrality *in*-degree | No significant relation |
|  |  |  |  |  | % of total ties within the sub-group of belonging | Significantly negative |
|  |  |  |  |  | % of total ties within the class | No significant relation |
|  |  |  |  |  | Connectedness to central students (Bonacich Index) | Significantly positive |
| Yang and Tang (2003) | Pearson correlation tests and OLS regressions | Cross sectional | 39 | Performance | *In*-degree centrality  in 3 types of networks : |  |
|  |  |  |  |  | Friendship | Significantly positive |
|  |  |  |  |  | Advice | Significantly positive |
|  |  |  |  |  | Adversarial | Significantly negative |
| Cho & al. (2007) | Pearson correlation tests | Longitudinal | 31 | Grades | Closeness centrality | Significantly positive |
|  |  |  |  |  | Centrality degree | Significantly positive |
| Zhang & al. (2008) | Pearson correlation tests  & hierarchical regression analysis | Cross sectional | 52 | Performance | Closeness centrality | Significantly positive |
| Obadi & al. (2010) | Spectral clustering and correlation | Cross sectional | 307 | Grades | Centrality (degree, closeness and betweenness) | Significantly positive for average grades |
| Hommes & al. (2012) | OLS regressions and structural equation models | Cross sectional | 301 | Knowledge test | Centrality degree and betweenness centrality in 3 types of networks | Significantly positive |
| Gašević & al. (2013) | OLS regressions and General Linear Model | Cross sectional | 505 | GPA | Centrality degree | No significant relation |
|  |  |  |  |  | Closeness centrality | Significantly positive |
|  |  |  |  |  | Betweenness centrality | No significant relation |
|  |  |  |  |  | Eccentricity | Significantly positive |
| **References** | **Statistical Tools** | **Perspective** | **N Valid** | **Dependent Variable(s)** | **Independent Variables(s)** | **Nature of Impact(s)**  **or of link(s)** |
| Mushtaq & al. (2016) | Spearman’s correlation  and OLS regression | Cross sectional | 182 | GPA | Centrality degree | Significantly positive |
|  |  |  |  |  | Betweenness centrality | Significantly positive |
|  |  |  |  |  | Closeness centrality | No significant relation |
| Zwolak & al. (2017) | Backward multiple logistic regression | Longitudinal | 220 | Persistence from one course toward another | *In*-degree centrality | Significantly positive |
|  |  |  |  |  | *Out*-degree centrality | Significantly positive |
|  |  |  |  |  | Closeness centrality | Significantly positive |
|  |  |  |  |  | Betweenness centrality | No significant relation |
|  |  |  |  |  | Eigenvector centrality | No significant relation |
| Vignery & Laurier (2020) | Univariate & multivariate regression models | Cross sectional | 120 | Score in a major course | Centrality *in*-degree  for friendship ties | Significantly positive in the univariate and in the multivariate models |
|  |  |  |  |  | Centrality *in*-degree  for strategic ties | Significantly positive only  in the univariate model |
|  |  |  |  |  | Centrality *out*-degree  for friendship ties | Significantly positive  only in the univariate model |
|  |  |  |  |  | Centrality *out*-degree  for strategic ties | No significant relation |
