# Supplementary material for: A methodology and theoretical taxonomy for centrality measures: What are the best centrality indicators for student networks?
Source: PLoS One. 2020 Dec 30;15(12):e0244377. doi: 10.1371/journal.pone.0244377 (PMC7773201; doi:10.1371/journal.pone.0244377)
Supplement: S3 Appendix — (DOCX) [file pone.0244377.s003.docx]

S3 Appendix. Resulting list of suitable centrality by using the function proper_centralities from the package CINNA in R©.

| Index | Author(s) (Year) | Short description | Direction of the edges used? | Number  of references^[[1]](#footnote-1)^ | Number  of references^[[2]](#footnote-2)^ |
| --- | --- | --- | --- | --- | --- |
| ***List of chosen centralities in our paper*** | | | | | |
| Eccentricity centrality | Jordan (1869) | See Appendix 3 |  | 1500 | 19 |
| Closeness centrality | Freeman (1979) |  |  | 18800 | 8480 |
| Residual closeness centrality | Dangalchev (2006) |  |  | 122 | 23 |
| Geodesic *k*-path centrality | Borgatti & Everett (2006) |  |  | 85 | 47 |
| Betweenness centrality | Freeman (1979) |  |  | 21500 | 15200 |
| Bottleneck centrality | Pržulj & al. (2004) |  |  | 203 | 4 |
| Eigenvector centrality | Bonacich (1972) |  |  | 6000 | 4470 |
| Hub score | Kleinberg (1999) |  |  | 7400 | 1600 |
| Authority score | Kleinberg (1999) |  |  | 10800 | 1890 |
| Page rank | Brin & Page (1998) |  |  | 26200 | 26200 |
| Cross-clique connectivity | Faghani & Nguyen (2013) |  |  | 82 | 5 |
| MNC - maximum neighborhood component | Lin & al. (2008) |  |  | 148000 | 126 |
| ***List of centralities not employed*** | | | | | |
| Alpha centrality | Bonacich & Lloyd (2001) | ‘*Generalized eigenvector measure of centrality*’. |  | 3170 | 181 |
| Barycenter centrality | Viswanath (2009) | Corresponds to the Freeman’s closeness centrality. |  | 63 | 4 |
| Bonacich power centralities of positions | Bonacich (1987) | The power of a node is computed according to the power of its adjacent nodes. |  | 7000 | 7 |
| Centroid value | Gräßler & al. (2012) | A node's distance from other nodes is weighted by the distance between all other nodes. |  | 24 | 2 |
| Closeness centrality | Dangalchev (2006) | Variant of closeness centrality. |  | 156 | 131 |
| Closeness centrality | Latora | Variant of closeness centrality. |  | 5510 | 1680 |
| Closeness vitality | Brandes (2005) | Change in the sum of distances between all node pairs when excluding a node. |  | 371 | 27 |
| Index | Author(s) (Year) | Short description | Direction of the edges used? | Number  of references₁ | Number  of references₂ |
| ClusterRank | Chen & al. (2013) | The influence of a node is computed by taking into account of its direct influence, of the influence of its neighbors, and of its clustering coefficient. |  | 547 | 547 |
| Communicability betweenness centrality | Estrada & al. (2009) | Variant of betweenness centrality. |  | 434 | 25 |
| Community centrality | Kalinka & Tomancak (2011) | Centrality based on the weighted communities a node belongs. |  | 120 | 11 |
| Current flow closeness centrality | Gräßler & al. (2012) | Closeness index specific to electrical currents. |  | 12 | 1 |
| Decay centrality | Coronicová Hurajová & al. (2018) | Centrality measure based on distance between nodes. |  | 2 | 2 |
| Diffusion degree | Pal & al. (2014) | Node centrality regarding cascade model of information diffusion. |  | 5020 | 9 |
| DMNC – density of MNC | Lin & al. (2008) | Variant of MNC. |  | 101000 | 101 |
| Entropy centrality | Ortiz-Arroyo & Hussain (2008) | The centrality of a node is related to its contribution to the entropy of the network. |  | 43 | 6 |
| Flow betweenness centrality | Freeman & al. (1991) | Variant of betweenness centrality. |  | 18100 | 338 |
| Group centrality | Everett & Borgatti (1999) | Centrality measures applied to groups and classes. |  | 28000 | 279 |
| Harary centrality | Hage & Harary (1995) | Variant of eccentricity. |  | 765 | 2 |
| Hubbell centrality (or Hubbell Index) | Hubbell (1965) | Variant of eigenvector centrality. |  | 2040 | 18 |
| Information centrality | Kelly (2017) | ‘*Relative change in the network efficiency*  *when a node is removed*’. |  | 85300 | 95 |
| K-core decomposition | Seidman (1983) | A node’s centrality is related to the connectedness of the region in which the node is located and connected to other nodes. |  | 766 | 437 |
| Katz centrality (Katz status index) | Katz (1953) | The centrality of a node is related to the number of its immediate neighbors and to its connections through these immediate neighbors. |  | 63400 | 1230 |
| Laplacian centrality | Qi & al. (2012) | Decrease in the networks’ Laplacian energy when a node is removed. |  | 1220 | 83 |
| Leverage centrality | Joyce & al. (2010) | *‘Centrality that considers the extent of connectivity of a node relative to the connectivity of its neighbors’.* |  | 4390 | 57 |
| Lin centrality | Lin (1976) | Variant of closeness centrality. |  | 133000 | 18 |
| Index | Author(s) (Year) | Short description | Direction of the edges used? | Number  of references₁ | Number  of references₂ |
| Load centrality | Goh & al. (2001) | Variant of betweenness centrality and specific to the analysis of flow structures. |  | 2120 | 43 |
| Lobby index (centrality) | Korn & al. (2009) | The centrality of a node is defined as the ‘*largest integer k such that x has at least k neighbors with a degree of at least k*’. |  | 1630 | 99 |
| Local bridging centrality | Macker (2016) | Classification of nodes according to their bridging characteristics. |  | 17300 | 4 |
| Markov centrality | White & Smyth (2003) | A node’s centrality score is defined by using random walks through the graph, i.e., by computing the mean first-passage time on the node. |  | 7620 | 49 |
| Radiality centrality | Wolfram Research (2014) | A node's distance from other nodes is weighted by the diameter of its reachable neighborhood. |  | 544 | 3 |
| Semi local centrality | Chen & al. (2012) | Centrality measure that identifies the influencers (i.e., nodes that lead to faster and wider spreading) in complex networks. |  | 21000 | 161 |
| Stress centrality | Shimbel (1953) | Variant of betweenness centrality. |  | 488 | 110 |
| Wiener index centrality | Wiener (1947) | Variant of closeness centrality. |  | 10800 | 1 |

1. Search made on Google Scholar on August 2019, based on the index name and on the name of the main author, both in ‘*all of the following words,* and *‘at any point in the paper’*. [↑](#footnote-ref-1)
2. Search made on Google Scholar on August 2019, based on the index name in ‘*exact expression’*, on the name of the main author in ‘*all of the following words’,* and *‘at any point in the paper’*. [↑](#footnote-ref-2)
