# Supplementary material for: A methodology and theoretical taxonomy for centrality measures: What are the best centrality indicators for student networks?
Source: PLoS One. 2020 Dec 30;15(12):e0244377. doi: 10.1371/journal.pone.0244377 (PMC7773201; doi:10.1371/journal.pone.0244377)
Supplement: S4 Appendix — (DOCX) [file pone.0244377.s004.docx]

S4 Appendix. Centrality measures: definitions.

1. ***Centralities based on the topological structure of the network***
   1. *Eccentricity centrality (in- & out-)*

The eccentricity, discovered by Camille Jordan in 1869 (Hage & Harary, 1995), represents the shortest path length between an individual and the farthest member with which he is connected in a social network (Gašević & al., 2013; Chin & al., 2014; de-Marcos & al., 2016; Lü & al., 2016; Saqr & al., 2018). Since it can be defined as the reciprocal of the maximum geodesic distance between a given node *i* and all other nodes in the network, individuals with low levels of eccentricity are considered to be more isolated and difficult to reach (Saqr & al., 2018a; Saqr & al., 2018b). The *out*- and *in*- eccentricity for a given node *i* are calculated as:

$$C_{Eo}\left( i \right)=\frac{1}{\max d(i,j)} (1)$$

$$C_{Ei}\left( i \right)=\frac{1}{\max d(j,i)} (2)$$

where max $d\left( i,j \right)$is the maximum geodesic distance between *i* and all other nodes towards he points to, and max $d\left( j,i \right)$is the maximum geodesic distance between all nodes *j* pointing *i*, with *i* ≠ *j*.

This measure is related to the access of valuable information diffused within a network (Gašević & al., 2013).

- 1. *Closeness centrality (in- & out-)*

The closeness centrality, introduced by Sabidussi in 1966 (Wasserman & Faust, 1994; Kolaczyk & Gábor, 2014) represents the distance or proximity of an individual to all other members in network (Cho & al., 2007; Ghali & al., 2012; Gašević & al., 2013; de-Marcos & al., 2016; Mersch, 2016; Mushtaq & al., 2016; Ghazzali & Ouellet, 2017; Zedan & Miller, 2017; Zwolak & al., 2017; Saqr & al., 2018). Freeman (1979) defined it as the reciprocal of the total geodesic distance between a given node *i* and all other nodes in the network (Borgatti & Everett, 2006; Zhang & al., 2011; Pfeffer & Carley, 2012; Chin & al., 2014; Kolaczyk & Gábor, 2014).

The closeness *out*- centrality of the node *i* represents the shortest paths between *i* and all other nodes in the network. It measures the required length to access every other nodes from a given one, i.e., the degree with which a node can easily reach other nodes. The closeness *out*- centrality for a given node *i* is calculated as:

$$C_{Co}\left( i \right)=\frac{1}{\sum_{j=1}^{N} d(i,j)} (3)$$

where *N* is the total number of nodes in the network, and $d\left( i,j \right)$the geodesic distance from *i* to *j*, with *i* ≠ *j*. When there is no path between *i* and *j*, we can use *N*, instead of the geodesic distance between *i* and *j* (Zwolak & al., 2017).

The closeness *in*- centrality of the node *i* represents the shortest paths between all other nodes and *i*. It measures the required length to access a given node from every other node in the network, i.e., the degree with which a node can be easily reached by other nodes. The closeness *in*- centrality for a given node *i* is calculated as:

$$C_{Ci}\left( i \right)=\frac{1}{\sum_{j=1}^{N} d(j,i)} (4)$$

where *N* is the total number of nodes in the network, and $d\left( j,i \right)$the geodesic distance from *j* to *i*, with *i* ≠ *j*. As for the closeness *out*- centrality, *N* can be used when there is no path between *j* and *i* (Zwolak & al., 2017).

The closeness centrality concerns the speed or efficiency with which the information will spread between nodes (Obadi & al., 2010; Song & al., 2015; Mersch, 2016), and members with high levels of closeness centrality have best vision of the information flow (Lü & al., 2016). Since central nodes are able to quickly contact other members (Landherr & al., 2010), they will enjoy efficient, easier and faster access to information, advice, resources and (academic) benefits in the network (Zhang & al., 2008; Ghali & al., 2012; Song & al., 2015; Mushtaq & al., 2016; Zedan & Miller, 2017; Zwolak & al., 2017; Vargas & al., 2018).

- 1. *Residual closeness centrality (in- & out-)*

The residual closeness centrality, proposed by Dangalchev (2006), measures the closeness after the removal of nodes or edges. It investigates the vulnerability or resistance of a graph after the suppression of nodes (Lü & al., 2016; Aytac & Berberler, 2017; Xu & al., 2018), i.e., after the removal of communication links. This index was used to investigate nodes centrality in several studies (e.g., Ashtiani & al., 2018; Xu & al., 2018; Song & al., 2015).

Dangalchev (2006) first proposed a definition of closeness for connected (i.e., graphs where all nodes are reachable) *and* disconnected graphs (i.e., graphs where there are unreachable nodes):

$$C\left( i \right)=\sum_{j=1}^{N} \frac{1}{2^{d(i,j)}} (5)$$

where *N* is the total number of nodes in the network, and $d\left( i,j \right)$the geodesic distance from *i* to *j*, with *i* ≠ *j*.

Then, the closeness of the node *k* is calculated as (Dangalchev, 2006):

$$C\left( k \right)=\sum_{i=1}^{N} \sum_{j=1}^{N} \frac{1}{2^{d_{k}(i,j)}} (6)$$

where *N* is the total number of nodes in the network, and $d_{k}\left( i,j \right)$the geodesic distance from *i* to *j* after removing the node *k*, with *i* ≠ *j ≠ k*. For directed graphs, we consider shortest paths either from (for the residual closeness *out*- centrality) or either to (for the residual closeness *in*- centrality) the nodes *i*.

A node with a small value of residual closeness centrality is significant for the network (Song & al., 2015), since its removal increases significantly the distance between other nodes.

- 1. *Betweenness centrality*

This index, proposed by Freeman (1977 & 1979), refers to the number of times that a node is located on the geodesic (i.e., shortest) path between two other nodes (Alahakoon & al., 2011; Pfeffer & Carley, 2012; Zhang & al., 2011; Gašević & al., 2013; Chin & al., 2014; Song & al., 2015; de-Marcos & al., 2016; Fouss & al., 2016; Lü & al., 2016; Mersch, 2016; Ghazzali & Ouellet, 2017; Zwolak & al., 2017; Vargas & al., 2018). This centrality measure reflects at what point a node is located in the middle of other nodes (Ghali & al., 2012; Kolaczyk & Gábor, 2014; Mushtaq & al., 2016). The betweenness centrality for a given node *i* is calculated as:

$$C_{B}\left( i \right)=\sum_{i\neq j\neq k} \frac{p_{jk(i)}}{p_{jk}} (7)$$

where $p_{jk}$ is the total number of geodesic paths from node *j* to node *k*, and $p_{jk}\left( i \right)$ is the number of geodesic paths from node *j* to node *k* that pass through *i*.

Actors with high levels of betweenness centrality connect other nodes and facilitate communication between other actors in the network (Cho & al, 2007; Obadi & al., 2010; Zedan & Miller, 2017; Saqr & al., 2018; Vargas & al., 2018). The betweenness centrality represents the access and control that a node has over the (novel) information and resources contained and flowing in a graph (Landherr & al., 2010; Pfeffer & Carley, 2012; Song & al., 2015; de-Marcos & al., 2016; Lü & al., 2016; Vargas & al., 2018), and therefore reflects his power or influence on other nodes (Ghali & al., 2012; Mersch, 2016; Zwolak & al., 2017).

- 1. *Geodesic k-path centrality (in- & out-)*

A variant of the betweenness centrality, the geodesic *k*-path centrality, proposed by Borgatti & Everett (2006), counts only the *shortest* paths up to length *k* emanating from/going towards a given node, and allows computing the number of individuals or neighbors that are reachable by the fastest path up to length *k* – i.e., that are on a geodesic path less than *k* away. This *k*-betweenness index bounds therefore the length or distance of the geodesic paths on which nodes are located (Alahakoon & al., 2011; Pfeffer & Carley, 2012; Ghazzali & Ouellet, 2017), since according to Borgatti & Everett (2006), “*long*” shortest paths are not necessarily relevant to spread information through the network. At the contrary, the geodesic *k*-path centrality informs about the reception and/or diffusion of local information (the smaller the *k* is and the more local the information are), instead of the spreading of information through the whole network (Ghazzali & Ouellet, 2017). In directed graphs, the geodesic *k*-path *out*- centrality of the node *i* corresponds to the number of individuals that are reachable by the node *i*, being on a geodesic path less than *k* away from *i*. The geodesic *k*-path *in*- centrality of the node *i* counts the number of neighbors that can reach *i* being on a geodesic path less than *k* away towards *i*.

- 1. *Bottleneck centrality (in- & out-)*

According to Obadi & al. (2010), bottlenecks are ‘*central nodes that provide the only connection between different parts of a network*’. Nodes with high bottleneck scores are therefore the most important ones in the network (Pržulj & al., 2004). This index, that represent the degree of confluence of edges through a given node (Idowu & al. 2004), is mostly used in biological studies (e.g., Idowu & al. 2004; Pržulj & al., 2004; Yu & al., 2007; Lin & al., 2008; Chin & al., 2014). Pržulj & al. (2004) proposed the following steps to compute bottlenecks inside a network:

- Step 1: For each node *i*, a tree $T_{i}$of shortest paths from *i* to all other nodes *j* in the graph is constructed. $T_{i}$contains $n_{i}$nodes, i.e., the number of nodes that are directly or indirectly connected to *i*.
- Step 2: We extract the nodes *k* for which more than $n_{i}/4$ paths from *i* to all the other nodes in $T_{i}$intersect in *k*. These nodes *k* are bottlenecks since many shortest paths from *i* to *j* in $T_{i}$ cross at *k*.
- Step 3: The same node *k* may be a bottleneck of several$T_{i}$, i.e., of different shortest path trees. The bottleneck score of *k* - ${BN}_{k}$ - equals the number of time that *k* appears on different$T_{i}$, i.e., represents the number of time that a node *k* is a bottleneck.

For directed graphs, we consider shortest paths either from (for the bottleneck *out*- centrality) or either to (for the bottleneck *in*- centrality) the nodes *i*.

1. ***Centralities based on the neighborhood***
   1. *Eigenvector prestige score*

As other centrality measures, eigenvector prestige score reflects the power, influence or importance of a node in a network (Batool & Niazi, 2014; de-Marcos & al., 2016; Mersch, 2016; Zwolak & al., 2017). The additional idea behind this centrality measure is that a node will be more prestigious or powerful if his neighbors are also central or well-connected (Landherr & al., 2010; Kolaczyk & Gábor, 2014; Fouss & al., 2016; Zedan & Miller, 2017). The computation proposed by Bonacich (1972) weights the number of connections that a node has by the own centrality of those connections (Fouss & al., 2016). In addition to the number of connections, the influence of those neighbors is therefore taken into account in order to determine the centrality of a node (Lü & al., 2016; Mersch, 2016; Ghazzali & Ouellet, 2017). Nodes with high scores of eigenvector prestige are connected to many members being themselves high-scoring nodes (Lü & al., 2016):

$$C_{EV}\left( i \right)=\alpha\sum_{j=1}^{N} e_{ij}C_{EV}\left( j \right) (8)$$

where *N* is the number of nodes in the network, $e_{ij}=1$ if there is an edge between *i* and *j* with *i* ≠ *j*, else $e_{ij}=0$, $C_{EV}\left( j \right)$ are the eigenvector scores of the nodes *j* that are connected to *i*, and $\alpha=\frac{1}{\lambda}$in which $\lambda$ is the largest eigenvalues of the adjacency matrix *E*.

- 1. *Page rank score*

Page Rank (Brin & Page, 1998; Page & al., 1999), a variant of the eigenvector centrality, and a measure of prestige, also quantifies the relative importance of a node within the network (de-Marcos & al., 2016). The iterative algorithm was initially developed to measure web pages centrality (Lü & al., 2016). Each page *p* is given a score proportional to the number of times one passes on *p*, having clicked on one of the links appearing on each page quoting *p*. Pages with high Page rank scores are pointed by many pages having themselves a high Page rank metric. In social networks (e.g., friendship networks), members that are cited by many individuals having a high degree of Page rank and a small *out*-degree (i.e., not citing many nodes) will see their own Page rank increase (Bruun & Brewe, 2013; Fouss & al., 2016) :

$$C_{PR}\left( i \right)= \sum_{j=1}^{N} \frac{e\left( j,i \right)C_{PR}(j)}{C_{Do}\left( j \right)} (9)$$

where$e\left( j,i \right)=1$ if there is an edge from *j* towards *i* with *i* ≠ *j*, else $e\left( j,i \right)=0$, $C_{PR}(j)$ is the Page rank score of the node *j*, and $C_{Do}\left( j \right)$ corresponds to the number of nominations that are made by *j*.

- 1. *Kleinberg's authority & hub centrality scores*

The authority & hub centrality scores (i.e., HITS algorithm or Hyperlink-Induced Topic Search) were proposed by Kleinberg (1999a, 1999b) and initially developed to assess web pages. Authorities are pages possessing many incoming links and containing high-quality information, and hubs are pages that point toward good authorities (Kiss & Bichler, 2008; Fouss & al., 2016; Lü & al., 2016). Therefore, “*a good hub should cite many good authorities, and a good authority should be cited by many good hubs*” (Fouss & al., 2016). Related to directed networks, as the eigenvector centrality or Page Rank, the authority score of a node reflects the importance of a node according to the number of important nodes – i.e., hubs - that point towards him. Then, a node will be important or central if he points towards other important nodes - i.e., if he possess a high hub score by pointing good authorities. The authority and the hub scores of node *i* at time *t* are^[[1]](#footnote-1)^ (Jarumaneeroj, 2014; Lü & al., 2016):

$${Aut}_{i}\left( t \right)=\sum_{j=1}^{N} e_{\left( j,i \right)}{Hub}_{j}\left( t-1 \right) (10)$$

where *N* is the number of nodes in the network, $e_{(j,i)}=1$ if there is an edge from *j* towards *i* with *j* ≠ *i*, else $e_{(j,i)}=0$, and ${Hub}_{j}$ are the hub scores of all *j* that point to *i*.

$${Hub}_{i}\left( t \right)=\sum_{j=1}^{N} e_{\left( i,j \right)}{Aut}_{j}\left( t \right) (11)$$

where *N* is the number of nodes in the network, $e_{(i,j)}=1$ if there is an edge from *i* towards *j* with *i* ≠ *j*, else $e_{(i,j)}=0$, and ${Aut}_{j}$are the authority scores of the nodes *j* that are pointed by *i*.

- 1. *MNC – The maximum neighborhood component (in- & out-)*

The open neighborhood ${GAdj}_{i}$of a node $i$in a graph $G$is the subgraph that is composed of the nodes adjacent to $i$ and of all edges connecting nodes adjacent to $i$. This neighborhood does not include $i$itself. The Maximum Neighborhood Component (MNC) was developed by Lin & al. (2008) and is mostly used in the study of biological networks (e.g., Flórez & al., 2010; Duran-Pinedo & al., 2011; Asgari & al., 2013; Ashtiani & al., 2018), even if it can also be used to identify central nodes on other types of graphs such as human networks (Lin & al., 2008). The centrality index concerns a target node and the topology properties, specifically the connectivity degree, of its neighborhood (Peng & al., 2015). The maximum neighborhood component for a given node *i* is calculated as (Lin & al., 2008; Zhang & al., 2011; Chin & al., 2014):

$${MNC}_{i}=N_{{GAdj}_{i}}^{max} (12)$$

where${MNC}_{i}$corresponds to the size of the maximum connected component of ${GAdj}_{i}$ - i.e., represents the number of nodes within ${GAdj}_{i}$ that are directly connected.

In directed graphs, for ‘*out*’, only the outgoing edges are followed, and for ‘*in*’ only the ingoing edges are taken into account.

- 1. *Cross-clique connectivity*

Formally, a clique consists of three or more nodes which have all possible edges present among themselves, without any other nodes outside the clique being also directly connected to all of the actors of this clique (Wasserman & Faust, 1994; Faghani & Nguyen, 2013; Mersch, 2016; Zedan & Miller, 2017). A same node *i* might belong to more than one clique. The cross-clique connectivity of the node *i* (${CC}_{i}$), also called cross-clique centrality, an index proposed by Faghani & Nguyen (2013) and that represents the level of connections of a node to different sub-communities in a network, counts the number of cliques to which *i* belongs (Ghazzali & Ouellet, 2017). Since ‘*cross-clique centrality measures how often a node connects distinct cliques*’ (Mersch, 2016), a node with a high value of $CC$possess a large influence in the graph, spreads and promotes more likely his ideas, facilitates the transfer of information between sub-communities in the network, and assures the cohesiveness of its clique (Mersch, 2016; Ghazzali & Ouellet, 2017; Zedan & Miller, 2017).

1. At the first iteration, the hub scores of all nodes are assigned to 1. [↑](#footnote-ref-1)
