# Supplementary material for: A methodology and theoretical taxonomy for centrality measures: What are the best centrality indicators for student networks?
Source: PLoS One. 2020 Dec 30;15(12):e0244377. doi: 10.1371/journal.pone.0244377 (PMC7773201; doi:10.1371/journal.pone.0244377)
Supplement: S5 Appendix — (DOCX) [file pone.0244377.s005.docx]

S5 Appendix. Imputation steps for the missing actors

According to Wasserman & Faust (1994), SNA methods require the complete recording of interactions between actors belonging to the studied network. In order to fulfill this condition, two methodological approaches can be used: the complete cases - respondent only approach (i.e., deleting the nominations corresponding to actors that do not complete the survey) or the imputation approach (i.e., imputing ties for the missing actors). The respondent only approach might produce more biased estimates than the imputation approach (e.g., Huisman, 2009; Wang & al., 2016; Gile & Handcock, 2017). Then, the nature of our graph (i.e., directed), the high proportion of missing actors (34.02%), and the fact that those actors were not missing at random - we observed significant differences of gender (χ²= 131.15, p-value = 0.000) and of curricula (χ² = 87.00, p-value = 0.000) between respondents and missing actors - lead us to select the Exponential Random Graph Models (i.e., ERGMs) as imputation method. Also, ERGMs give better performances than simpler imputation procedures (Huisman & al., 2018).

As in regression models, where the goal is to model a dependent variable based on potential predictors, ERGMs can model the probability of a network structure - i.e., the pattern of edges within the graph - as a function of statistics (e.g., the number of ties within the entire network, the number of ties between specific groups of nodes ...) that are computed on the network (Robins & al., 2004; Hunter & al., 2008; Morris & al., 2008; Goodreau & al., 2009; Lusher & al., 2013). The conditional log-odds of tie between pairs of nodes (i.e., the output of ERGMs) can then be used to impute (or not) edges between those pairs of nodes.

The formula, exactly transcribed from Handcock & al., 2008 (see the paper for further details), that computes the probability of edges between nodes is:

$$P\left( \boldsymbol{Y}=y | \boldsymbol{X} \right)=exp\left[ \theta^{T}g\left( y,\boldsymbol{X} \right) \right]/k(\theta)$$

The model computes the probability of the set of relations **Y** (with *y* being a particular set of links), according **X**, the matrix of the nodes’ characteristics. $g\left( y,X \right)$is a vector of statistics that are computed on the graph, $\theta$ is the vector of coefficients for each tested nodes’ characteristics, and $k(\theta)$ is a normalized constant.

The table 1 summarizes the final model that we used to impute the ties on the missing actors:

| Effects | Estimate | Std. Error | p-value |
| --- | --- | --- | --- |
| Number of edges in the network | -5.48 | 0.22 | <0.001 |
| Node mixing by gender | -0.07 | 0.03 | 0.05 |
| Node mixing by curriculum | -0.13 | 0.02 | <0.001 |
| Homophily : Law | 1.83 | 0.07 | <0.001 |
| Homophily : Economy | 2.72 | 0.11 | <0.001 |
| Homophily : Management | 3.98 | 0.11 | <0.001 |
| Homophily : Literature, philosophy & history | 4.17 | 0.13 | <0.001 |
| Homophily : Communication, political & social sciences | 1.82 | 0.13 | <0.001 |
| Homophily : Translation & interpretation studies | 3.90 | 0.16 | <0.001 |

In order to diagnose our model, first we computed the percentage of ties that the model allowed recovering among the respondents (i.e., even if, for further analyses, we conserved the original ties for the respondents, and that we use the imputed links only for the non respondents). The final model that was used allowed recovering 39.87% of ties among the respondents.

Then, since the effect of node mixing by curricula was significant, we compared the proportion of *out*-degree (i.e., nominations of friends) within and outside same curricula, between the respondents and missing actors. Results show that 84.93% of respondents nominated friends from same curriculum and that 71.18% of ties were imputed within the same curriculum for the missing actors (see table 2). Even if the compared proportions between respondents and missing actors were significantly different (χ² = 101.75; *p-value* <0.0001), as showed in table 1, the model takes into account of the homophily effect by curriculum when imputing ties. Moreover, we do not know the curriculum for 5.73% of the missing actors, which might be increase the proportion of nominations within same curriculum for those non respondents.

Table 2. Proportion of nominations within and outside same curricula, between the respondents and missing actors.

|  | % of ties within the same curriculum | % of ties within a different curriculum | % of missing curriculum |
| --- | --- | --- | --- |
| Respondents | 84.93 | 15.07 | 0.00 |
| Missing actors | 71.18 | 23.09 | 5.73 |
| Total | 81.47 | 17.09 | 1.44 |
