# Supplementary material for: A methodology and theoretical taxonomy for centrality measures: What are the best centrality indicators for student networks?
Source: PLoS One. 2020 Dec 30;15(12):e0244377. doi: 10.1371/journal.pone.0244377 (PMC7773201; doi:10.1371/journal.pone.0244377)
Supplement: S6 Appendix — (DOCX) [file pone.0244377.s006.docx]

S6 Appendix. Results of the PCA for the respondents only (n = 574)

**Table 1. Correlations between the eighteen centrality measures.**

In bold front: coefficients for which the p-value is ≤ 0.05

|  | Ecc *in-* | Ecc *out-* | Clos *in-* | Clos *out-* | Res clos  *in-* | Res clos *out-* | Between | *k*-path *in-* | *k*-path *out-* | Bottle *in-* | Bottle *out-* | Eigen. | Page rank | Authority | Hub | MNC *in-* | MNC *out-* | Clique |
| --- | --- | --- | --- | --- | --- | --- | --- | --- | --- | --- | --- | --- | --- | --- | --- | --- | --- | --- |
| Ecc *in-* | 1.00 |  |  |  |  |  |  |  |  |  |  |  |  |  |  |  |  |  |
| Ecc *out-* | **.18** | 1.00 |  |  |  |  |  |  |  |  |  |  |  |  |  |  |  |  |
| Clos *in-* | **.93** | **.15** | 1.00 |  |  |  |  |  |  |  |  |  |  |  |  |  |  |  |
| Clos *out-* | **.10** | **.90** | .03 | 1.00 |  |  |  |  |  |  |  |  |  |  |  |  |  |  |
| Res clos *in-* | **.78** | **.27** | **.79** | **.18** | 1.00 |  |  |  |  |  |  |  |  |  |  |  |  |  |
| Res clos *out-* | **.50** | **.53** | **.45** | **.50** | **.66** | 1.00 |  |  |  |  |  |  |  |  |  |  |  |  |
| Between | **.27** | **.34** | **.29** | **.36** | **.42** | **.49** | 1.00 |  |  |  |  |  |  |  |  |  |  |  |
| *k*-path *in-* | **.76** | **.26** | **.75** | **.18** | **.98** | **.65** | **.39** | 1.00 |  |  |  |  |  |  |  |  |  |  |
| *k*-path *out-* | **.47** | **.48** | **.42** | **.44** | **.63** | **.98** | **.45** | **.64** | 1.00 |  |  |  |  |  |  |  |  |  |
| Bottle *in-* | **.08** | .06 | **.08** | .03 | **.18** | **.16** | **.08** | **.19** | **.16** | 1.00 |  |  |  |  |  |  |  |  |
| Bottle *out-* | **.09** | .06 | **.08** | .03 | **.19** | **.19** | **.08** | **.21** | **.19** | **.81** | 1.00 |  |  |  |  |  |  |  |
| Eigenvector | **.28** | .00 | **.39** | -.04 | **.32** | **.23** | .06 | **.29** | **.22** | .05 | .06 | 1.00 |  |  |  |  |  |  |
| Page rank | **.43** | .05 | **.39** | .02 | **.59** | **.20** | **.23** | **.55** | **.14** | **.18** | **.17** | **.10** | 1.00 |  |  |  |  |  |
| Authority | **.28** | .00 | **.38** | -.04 | **.32** | **.24** | .06 | **.28** | **.23** | .03 | .04 | **.98** | **.09** | 1.00 |  |  |  |  |
| Hub | **.26** | .00 | **.35** | -.03 | **.29** | **.27** | .05 | **.26** | **.25** | .06 | .06 | **.89** | **.08** | **.90** | 1.00 |  |  |  |
| MNC *in-* | **.55** | **.18** | **.53** | **.12** | **.76** | **.58** | **.21** | **.68** | **.51** | **.16** | **.16** | **.37** | **.56** | **.37** | **.33** | 1.00 |  |  |
| MNC *out-* | **.49** | **.26** | **.47** | **.17** | **.64** | **.68** | **.18** | **.58** | **.60** | **.16** | **.17** | **.36** | **.45** | **.36** | **.42** | **.83** | 1.00 |  |
| Clique | **.49** | **.15** | **.52** | **.08** | **.69** | **.62** | **.25** | **.62** | **.56** | **.12** | **.12** | **.56** | **.40** | **.59** | **.61** | **.81** | **.81** | 1.00 |

**Table 2. KMO & Bartletts test**

| Kaiser-Meyer-Olkin Measure of Sampling Adequacy | | 0,78 |
| --- | --- | --- |
| Bartletts test of Sphericity | Chi-Square χ² | 14260.61 |
|  | P-value | 0.000 |

**Table 3. Sum of the square correlation coefficients between a variable and each factorial axis.**

| Variables | Extraction |
| --- | --- |
| Ecc *in-* | .86 |
| Ecc *out-* | .80 |
| Clos *in-* | .90 |
| Clos *out-* | .82 |
| Res clos *in-* | .93 |
| Res clos *out-* | .86 |
| Between | .45 |
| *k*-path *in-* | .88 |
| *k*-path *out-* | .76 |
| Bottle *in-* | .90 |
| Bottle *out-* | .90 |
| Eigenvector | .95 |
| Page rank | .56 |
| Authority | .95 |
| Hub | .91 |
| MNC *in-* | .88 |
| MNC *out-* | .88 |
| Clique | .87 |

**Table 4. Percentages of variance retained by the first five factorial axes.**

| Components | Initial Eigenvalues | | |
| --- | --- | --- | --- |
|  | Total | % of Variance | Cumulative % |
| 1 | 7.61 | 42.29 | 42.29 |
| 2 | 2.76 | 15.36 | 57.65 |
| 3 | 1.93 | 10.75 | 68.40 |
| 4 | 1.71 | 9.51 | 77.91 |
| 5 | 1.05 | 5.81 | 83.72 |

**Table 5. Results of the Varimax rotation: correlation of each variable on the factorial axis on which the saturation is the highest.**

|  | Components | | | | |
| --- | --- | --- | --- | --- | --- |
| Centrality Indices | 1 | 2 | 3 | 4 | 5 |
| Closeness centrality (*out-*) | 0.90 |  |  |  |  |
| Residual closeness centrality (*out-*) | 0.70 |  |  |  |  |
| Eccentricity centrality (*out-*) | 0.89 |  |  |  |  |
| Geodesic *k*-path centrality (*out-*) | 0.67 |  |  |  |  |
| Betweenness | .052 |  |  |  |  |
| Kleinberg's authority centrality scores |  | .96 |  |  |  |
| Eigenvector prestige score |  | .95 |  |  |  |
| Kleinberg's hub centrality scores |  | .93 |  |  |  |
| Geodesic *k*-path Centrality (*in-*) |  |  | .76 |  |  |
| Residual closeness centrality (*in-*) |  |  | .76 |  |  |
| Eccentricity centrality (*in-*) |  |  | 0.87 |  |  |
| Closeness centrality (*in-*) |  |  | 0.89 |  |  |
| MNC - maximum neighborhood component (*out-*) |  |  |  | .85 |  |
| MNC - maximum neighborhood component (*in-*) |  |  |  | .84 |  |
| Cross-clique connectivity |  |  |  | .74 |  |
| Page rank |  |  |  | .55 |  |
| Bottleneck centrality (*in-*) |  |  |  |  | 0.94 |
| Bottleneck centrality (*out-*) |  |  |  |  | 0.94 |
