# Supplementary material for: A methodology and theoretical taxonomy for centrality measures: What are the best centrality indicators for student networks?
Source: PLoS One. 2020 Dec 30;15(12):e0244377. doi: 10.1371/journal.pone.0244377 (PMC7773201; doi:10.1371/journal.pone.0244377)
Supplement: S7 Appendix — (DOCX) [file pone.0244377.s007.docx]

S7 Appendix.

**Table 1.** **Distribution of the data: Skewness and Kurtosis measures for the respondents’ network and the augmented network.**

| Network | | n=574 | n=870 |
| --- | --- | --- | --- |
| Eccentricity (*in*-) | Skewness | 0.63 | -1.65 |
|  | Kurtosis | -1.24 | 1.34 |
| Eccentricity (*out*-) | Skewness | 1.02 | -.98 |
|  | Kurtosis | -0.21 | -.76 |
| Closeness (*in*-) | Skewness | 1.14 | -1.94 |
|  | Kurtosis | -0.02 | 1.78 |
| Closeness (*out*-) | Skewness | 1.61 | -1.12 |
|  | Kurtosis | 1.60 | -.74 |
| Residual closeness (*in*-) | Skewness | 1.05 | .27 |
|  | Kurtosis | 0.41 | -.42 |
| Residual closeness (*out*-) | Skewness | 1.05 | .46 |
|  | Kurtosis | 0.37 | -.48 |
| Geodesic *k*-path (*in*-) | Skewness | 1.19 | .99 |
|  | Kurtosis | 0.70 | .96 |
| Geodesic *k*-path (*out*-) | Skewness | 1.35 | 1.17 |
|  | Kurtosis | 1.25 | 1.36 |
| **Betweenness** | Skewness | 4.49 | 2.75 |
|  | Kurtosis | 23.83 | 12.06 |
| **Bottleneck (*in*-)** | Skewness | 5.63 | 4.57 |
|  | Kurtosis | 46.23 | 30.35 |
| **Bottleneck (*out*-)** | Skewness | 4.97 | 5.69 |
|  | Kurtosis | 38.40 | 51.69 |
| **Eigenvector prestige score** | Skewness | 6.91 | 7.58 |
|  | Kurtosis | 49.76 | 62.05 |
| **Hub score** | Skewness | 7.81 | 9.04 |
|  | Kurtosis | 64.93 | 87.90 |
| **Authority score** | Skewness | 7.10 | 7.96 |
|  | Kurtosis | 52.21 | 68.14 |
| **Page rank** | Skewness | 1.26 | 2.70 |
|  | Kurtosis | 1.96 | 11.77 |
| **Cross-clique connectivity** | Skewness | 3.40 | 2.69 |
|  | Kurtosis | 14.51 | 8.52 |
| **MNC (*in*-)** | Skewness | 1.46 | 1.80 |
|  | Kurtosis | 2.97 | 4.63 |
| **MNC (*out*-)** | Skewness | 1.58 | 2.18 |
|  | Kurtosis | 3.85 | 6.91 |

**Table 2. Spearman correlations between the eighteen centrality measures for the augmented network.**

In bold front: coefficients for which the p-value is ≤ 0.05 (including those for which the p-value is ≤ 0.01)

|  | Ecc *in-* | Ecc *out-* | Clos *in-* | Clos *out-* | Res clos  *in-* | Res clos *out-* | Be-tween | *k*-path *in-* | *k*-path *out-* | Bottle *in-* | Bottle *out-* | Eigen. | Page rank | Authority | Hub | MNC *in-* | MNC *out-* | Clique |
| --- | --- | --- | --- | --- | --- | --- | --- | --- | --- | --- | --- | --- | --- | --- | --- | --- | --- | --- |
| Ecc *in-* | 1.00 |  |  |  |  |  |  |  |  |  |  |  |  |  |  |  |  |  |
| Ecc *out-* | **.07** | 1.00 |  |  |  |  |  |  |  |  |  |  |  |  |  |  |  |  |
| Clos *in-* | **.12** | **-.19** | 1.00 |  |  |  |  |  |  |  |  |  |  |  |  |  |  |  |
| Clos *out-* | **-.17** | **.33** | **-.16** | 1.00 |  |  |  |  |  |  |  |  |  |  |  |  |  |  |
| Res clos *in-* | **-.07** | **.15** | **.76** | **.21** | 1.00 |  |  |  |  |  |  |  |  |  |  |  |  |  |
| Res clos *out-* | -.04 | **.30** | -.03 | **.93** | **.36** | 1.00 |  |  |  |  |  |  |  |  |  |  |  |  |
| Between | .02 | **.36** | **.25** | **.59** | **.63** | **.73** | 1.00 |  |  |  |  |  |  |  |  |  |  |  |
| *k*-path *in-* | -.04 | **.14** | **.73** | **.21** | **.99** | **.36** | **.63** | 1.00 |  |  |  |  |  |  |  |  |  |  |
| *k*-path *out-* | -.03 | **.30** | -.04 | **.92** | **.35** | **.99** | **.72** | **.35** | 1.00 |  |  |  |  |  |  |  |  |  |
| Bottle *in-* | -.04 | **-.08** | **-.07** | -.01 | **-.08** | -.01 | **-.09** | **-.07** | -.01 | 1.00 |  |  |  |  |  |  |  |  |
| Bottle *out-* | -.04 | **-.13** | .01 | -.03 | -.01 | -.04 | -.06 | .00 | -.04 | **.30** | 1.00 |  |  |  |  |  |  |  |
| Eigenvector | -.04 | **.14** | **.59** | **.17** | **.82** | **.33** | **.55** | **.81** | **.33** | -.05 | -.02 | 1.00 |  |  |  |  |  |  |
| Page rank | **.10** | **.10** | **.68** | .00 | **.70** | **.14** | **.46** | **.70** | **.13** | .03 | .04 | **.58** | 1.00 |  |  |  |  |  |
| Authority | -.03 | **.13** | **.42** | **.30** | **.68** | **.41** | **.47** | **.70** | **.40** | -.04 | .02 | **.65** | **.35** | 1.00 |  |  |  |  |
| Hub | -.02 | **.31** | .05 | **.70** | **.38** | **.80** | **.60** | **.39** | **.80** | -.02 | .00 | **.40** | **.22** | **.48** | 1.00 |  |  |  |
| MNC *in-* | .09 | **.15** | **.35** | **.22** | **.52** | **.32** | **.37** | **.53** | **.33** | .02 | .06 | **.51** | **.52** | **.55** | **.37** | 1.00 |  |  |
| MNC *out-* | .03 | **.37** | **.07** | **.51** | **.31** | **.58** | **.44** | **.32** | **.58** | **.16** | .06 | **.32** | **.38** | **.34** | **.61** | **.59** | 1.00 |  |
| Clique | .01 | **.25** | **.28** | **.55** | **.61** | **.68** | **.63** | **.62** | **.68** | .02 | .00 | **.55** | **.45** | **.62** | **.64** | **.73** | **.65** | 1.00 |
