# Supplementary material for: A methodology and theoretical taxonomy for centrality measures: What are the best centrality indicators for student networks?
Source: PLoS One. 2020 Dec 30;15(12):e0244377. doi: 10.1371/journal.pone.0244377 (PMC7773201; doi:10.1371/journal.pone.0244377)
Supplement: S1 File — (DOCX) [file pone.0244377.s008.docx]

**References appearing only in the supplementary files.**

Alahakoon, T., Tripathi, R., Kourtellis, N., Simha, R., & Iamnitchi, A. (2011). *K-path centrality: A new centrality measure in social networks*. In Proceedings of the 4th workshop on social network systems (pp 1-6).

Aytac, A., & Berberler, Z. N. O. (2017). Residual closeness for helm and sunflower graphs. *TWMS Journal of Applied and Engineering Mathematics*, *7*(2), 209.

Bonacich, P. (1972). Factoring and weighting approaches to status scores and clique identification. *Journal of mathematical sociology*, *2*(1), 113-120.

Bonacich, P. (1987). Power and centrality: A family of measures. *American journal of sociology*, *92*(5), 1170-1182.

Bonacich, P., & Lloyd, P. (2001). Eigenvector-like measures of centrality for asymmetric relations. *Social networks*, *23*(3), 191-201.

Brandes, U. (2005). *Network analysis: methodological foundations* (Vol. 3418). Springer Science & Business Media.

Chen, D., Lü, L., Shang, M. S., Zhang, Y. C., & Zhou, T. (2012). Identifying influential nodes in complex networks. *Physica a: Statistical mechanics and its applications*, *391*(4), 1777-1787.

Chen, D. B., Gao, H., Lü, L., & Zhou, T. (2013). Identifying influential nodes in large-scale directed networks: the role of clustering. *PloS one*, *8*(10), e77455.

Coronicová Hurajová, J., Gago Álvarez, S., & Madaras, T. (2018). On decay centrality in graphs. *Mathematica scandinavica*, *123*(1), 39-50.

Dangalchev, C. (2006). Residual closeness in networks. *Physica A: Statistical Mechanics and its Applications*, *365*(2), 556-564.

Estrada, E., Higham, D. J., & Hatano, N. (2009). Communicability betweenness in complex networks. *Physica A: Statistical Mechanics and its Applications*, *388*(5), 764-774.

Everett, M. G., & Borgatti, S. P. (1999). The centrality of groups and classes. *The Journal of mathematical sociology*, *23*(3), 181-201.

Freeman, L. C. (1977). A set of measures of centrality based on betweenness. *Sociometry*, 35-41.

Gile, K., & Handcock, M. S. (2006) *Model-based assessment of the impact of missing data on inference for networks* (Seattle, University of Washington).

Gile, K. J., & Handcock, M. S. (2017) Analysis of networks with missing data with application to the National Longitudinal Study of Adolescent Health, *Journal of the Royal Statistical Society: Series C (Applied Statistics)*, *66*(3), 501-519.

Goodreau, S. M., Handcock, M. S., Hunter, D. R., Butts, C. T., & Morris, M. (2008). A statnet Tutorial. *Journal of statistical software*, *24*(9), 1.

Goh, K. I., Kahng, B., & Kim, D. (2001). Universal behavior of load distribution in scale-free networks. *Physical Review Letters*, *87*(27), 278701.

Hage, P., & Harary, F. (1995). Eccentricity and centrality in networks. *Social networks*, *17*(1), 57-63.

Hubbell, C. H. (1965). An input-output approach to clique identification. *Sociometry*, 377-399.

Huisman, M., Krause, R. W., Alhajj, R., & Rokne, J. (2018). Imputation of Missing Network Data.

Joyce, K. E., Laurienti, P. J., Burdette, J. H., & Hayasaka, S. (2010). A new measure of centrality for brain networks. *PloS one*, *5*(8), e12200.

Jarumaneeroj, P. (2014). *An economics study of container ports in the global network of container shipping* (Doctoral dissertation, Georgia Institute of Technology).

Katz, L. (1953). A new status index derived from sociometric analysis. *Psychometrika*, *18*(1), 39-43.

Kelly, S. T. (2017). *A Bioinformatics Approach to Synthetic Lethal Interactions in Cancer with Gene Expression Data* (Doctoral dissertation). University of Otago.

Kelly, S. T. (2019). *TomKellyGenetics/info.centrality: an R implementation of information centrality using igraph*. Retrieved from <https://rdrr.io/github/TomKellyGenetics/info.centrality/>

Kleinberg, J. M. (1999a). Authoritative sources in a hyperlinked environment. *Journal of the ACM (JACM)*, *46*(5), 604-632.

Kleinberg, J. M. (1999b). Hubs, authorities, and communities. *ACM computing surveys (CSUR)*, *31*(4es), 5.

Korn, A., Schubert, A., & Telcs, A. (2009). Lobby index in networks. *Physica A: Statistical Mechanics and its Applications*, *388*(11), 2221-2226.

Lin, N. (1976). *Foundations of social research*. McGraw-Hill Companies.

Lusher, D., Koskinen, J., & Robins, G. (Eds.). (2013). *Exponential random graph models for social networks: Theory, methods, and applications*. Cambridge University Press.

Morris, M., Handcock, M. S., & Hunter, D. R. (2008). Specification of exponential-family random graph models: terms and computational aspects. *Journal of statistical software*, *24*(4), 1548.

Ortiz-Arroyo, D., & Hussain, D. A. (2008, December). An information theory approach to identify sets of key players. In *European Conference on Intelligence and Security Informatics* (pp. 15-26). Springer, Berlin, Heidelberg.

Mastrobuoni, G., & Patacchini, E. (2012). Organized crime networks: An application of network analysis techniques to the American mafia. *Review of Network Economics*, *11*(3).

Pal, S. K., Kundu, S., & Murthy, C. A. (2014). Centrality measures, upper bound, and influence maximization in large scale directed social networks. *Fundamenta Informaticae*, *130*(3), 317-342.

Qi, X., Fuller, E., Wu, Q., Wu, Y., & Zhang, C. Q. (2012). Laplacian centrality: A new centrality measure for weighted networks. *Information Sciences*, *194*, 240-253.

Rdocumentation. (2017). *centiserve v1.0.0*. Retrieved September 17, 2019 from https://www.rdocumentation.org/packages/centiserve/versions/1.0.0

rdrr.io. (2019). *centiserve: Find Graph Centrality Indices*. Retrieved September 17, 2019 from <https://rdrr.io/cran/centiserve/>

Seidman, S. B. (1983). Network structure and minimum degree. *Social networks*, *5*(3), 269-287.

Shimbel, A. (1953). Structural parameters of communication networks. *The bulletin of mathematical biophysics*, *15*(4), 501-507.

Viswanath, M. (2009). *Ontology-based automatic text summarization* (Doctoral dissertation). uga.

Wang, C., Butts, C. T., Hipp, J. R., Jose, R., & Lakon, C. M. (2016) Multiple imputation for missing edge data: A predictive evaluation method with application to Add Health. *Social networks*, *45*, 89-98.

White, S., & Smyth, P. (2003, August). Algorithms for estimating relative importance in networks. In *Proceedings of the ninth ACM SIGKDD international conference on Knowledge discovery and data mining* (pp. 266-275). ACM.

Wiener, H. (1947). Structural determination of paraffin boiling points. *Journal of the American Chemical Society*, *69*(1), 17-20.

Wolfram Research. (2015). *Radiality Centrality*. Retrieved from http://reference.wolfram.com/language/ref/RadialityCentrality.html

Zhang, X., & Venkatesh, V. (2013). Explaining employee job performance: The role of online and offline workplace communication networks. Mis Quarterly, 695-722.
