# Supplementary material for: A methodology and theoretical taxonomy for centrality measures: What are the best centrality indicators for student networks?
Source: PLoS One. 2020 Dec 30;15(12):e0244377. doi: 10.1371/journal.pone.0244377 (PMC7773201; doi:10.1371/journal.pone.0244377)
Supplement: Attachment — Submitted filename: PONE-D-20-06951_reviewer.pdf [file pone.0244377.s009.pdf]

PLOS ONE

A methodology and a theoretical taxonomy for centrality measures: What are the best centrality indicators for student networks?

--Manuscript Draft--

**Manuscript Number:**	PONE-D-20-06951
**Article Type:**	Research Article
**Full Title:**	A methodology and a theoretical taxonomy for centrality measures: What are the best centrality indicators for student networks?
**Short Title:**	What are the best centrality indicators for student networks?
**Corresponding Author:**	Kristel Vignery, M.D.<br>Universite Saint-Louis<br>Brussels, BELGIUM
**Keywords:**	
**Abstract:**	In social network analysis (SNA) several measures were developed to assess a nodes' centrality in a graph. Among these measures, we find connectivity or degree-based measures, geodesic distance-based indices, geodesic path-based scores and centrality measures related to a node's neighborhood. Moreover, studies that investigate networks use mostly degree, closeness and/or betweenness centralities, even if other measures might be valid candidates for representing and understanding these networks. The main contribution of this paper is the development of a methodology that allows comparing and validating centrality indices when studying a particular network of interest. The proposed methodology integrates the following steps: the choice of the centrality measures regarding the network of interest; the development of a theoretical taxonomy of these measures; the identification, by means of Principal Component Analysis (PCA), of latent dimensions of centrality within the network of interest; the verification of the proposed taxonomy of centrality measures; and the identification of the centrality measures that best represent the network of interest. Subsequently, we applied the proposed methodology on an existing graph of interest, i.e., on a real student network. We chose eighteen centrality measures that were developed in SNA, we thoroughly defined them, and we proposed a theoretical taxonomy of these eighteen measures. The PCA shows the emergence of six latent dimensions of centrality within the student network and, most importantly, shows that most of the centrality indices saturate on the same categories as those proposed by the theoretical taxonomy. Additionally, the results suggest that other indices than the most frequently applied might be more relevant for research on student networks.
**Order of Authors:**	Kristel Vignery, M.D.
	Wim Laurier
**Additional Information:**	
**Question**	**Response**
**Financial Disclosure**<br><br>Enter a financial disclosure statement that describes the sources of funding for the work included in this submission. Review the submission guidelines for detailed requirements. View published research articles from *PLOS ONE* for specific examples.<br><br>This statement is required for submission and **will appear in the published article** if the submission is accepted. Please make	The author(s) received no specific funding for this work.

Powered by Editorial Manager® and ProduXion Manager® from Aries Systems Corporation

sure it is accurate.<br><br>**Unfunded studies**<br>Enter: *The author(s) received no specific funding for this work.*<br><br>**Funded studies**<br>Enter a statement with the following details:<br>• Initials of the authors who received each award<br>• Grant numbers awarded to each author<br>• The full name of each funder<br>• URL of each funder website<br>• Did the sponsors or funders play any role in the study design, data collection and analysis, decision to publish, or preparation of the manuscript?<br>• **NO** - Include this sentence at the end of your statement: *The funders had no role in study design, data collection and analysis, decision to publish, or preparation of the manuscript.*<br>• **YES** - Specify the role(s) played.<br><br>* typeset	
**Competing Interests**<br><br>Use the instructions below to enter a competing interest statement for this submission. On behalf of all authors, disclose any competing interests that could be perceived to bias this work—acknowledging all financial support and any other relevant financial or non-financial competing interests.<br><br>This statement **will appear in the published article** if the submission is accepted. Please make sure it is accurate. View published research articles from *PLOS ONE* for specific examples.	The authors have declared that no competing interests exist.

**NO authors have competing interests**<br><br>Enter: *The authors have declared that no competing interests exist.*<br><br>**Authors with competing interests**<br><br>Enter competing interest details beginning with this statement:<br><br>*I have read the journal's policy and the authors of this manuscript have the following competing interests: [insert competing interests here]*<br><br>* typeset	
**Ethics Statement**<br><br>Enter an ethics statement for this submission. This statement is required if the study involved:<br><br>• Human participants<br>• Human specimens or tissue<br>• Vertebrate animals or cephalopods<br>• Vertebrate embryos or tissues<br>• Field research<br><br>Write "N/A" if the submission does not require an ethics statement.<br><br>General guidance is provided below. Consult the submission guidelines for detailed instructions. **Make sure that all information entered here is included in the Methods section of the manuscript.**	The study concerns human subjects<br>The juridic department of the USL-B approved the study<br>The survey contained all the required information for an informed consent and the survey was not mandatory<br>the data were anonymized before the analyses

Powered by Editorial Manager® and ProduXion Manager® from Aries Systems Corporation

**Format for specific study types**<br><br>**Human Subject Research (involving human participants and/or tissue)**<br>• Give the name of the institutional review board or ethics committee that approved the study<br>• Include the approval number and/or a statement indicating approval of this research<br>• Indicate the form of consent obtained (written/oral) or the reason that consent was not obtained (e.g. the data were analyzed anonymously)<br><br>**Animal Research (involving vertebrate animals, embryos or tissues)**<br>• Provide the name of the Institutional Animal Care and Use Committee (IACUC) or other relevant ethics board that reviewed the study protocol, and indicate whether they approved this research or granted a formal waiver of ethical approval<br>• Include an approval number if one was obtained<br>• If the study involved *non-human primates*, add *additional details* about animal welfare and steps taken to ameliorate suffering<br>• If anesthesia, euthanasia, or any kind of animal sacrifice is part of the study, include briefly which substances and/or methods were applied<br><br>**Field Research**<br><br>Include the following details if this study involves the collection of plant, animal, or other materials from a natural setting:<br>• Field permit number<br>• Name of the institution or relevant body that granted permission	
**Data Availability**<br><br>Authors are required to make all data underlying the findings described fully available, without restriction, and from the time of publication. PLOS allows rare exceptions to address legal and ethical concerns. See the PLOS Data Policy and FAQ for detailed information.	No - some restrictions will apply

A Data Availability Statement describing where the data can be found is required at submission. Your answers to this question constitute the Data Availability Statement and **will be published in the article**, if accepted.<br><br>**Important:** Stating 'data available on request from the author' is not sufficient. If your data are only available upon request, select 'No' for the first question and explain your exceptional situation in the text box.<br><br>Do the authors confirm that all data underlying the findings described in their manuscript are fully available without restriction?	
**Describe where the data may be found in full sentences. If you are copying our sample text, replace any instances of XXX with the appropriate details.**<br><br>• If the data are **held or will be held in a public repository**, include URLs, accession numbers or DOIs. If this information will only be available after acceptance, indicate this by ticking the box below. For example: *All XXX files are available from the XXX database (accession number(s) XXX, XXX.*).<br>• If the data are all contained **within the manuscript and/or Supporting Information files**, enter the following: *All relevant data are within the manuscript and its Supporting Information files.*<br>• If neither of these applies but you are able to provide **details of access elsewhere**, with or without limitations, please do so. For example:<br><br>*Data cannot be shared publicly because of [XXX]. Data are available from the XXX Institutional Data Access / Ethics Committee (contact via XXX) for researchers who meet the criteria for access to confidential data.*<br><br>*The data underlying the results presented in the study are available from (include the name of the third party*	Original data cannot be shared publicly because they are not anonymous. We will need the journal's help to make our data available.

and contact information or URL).<br>• This text is appropriate if the data are owned by a third party and authors do not have permission to share the data.<br><br>* typeset	
Additional data availability information:	

Click here to download Manuscript Taxonomy_Main text.docx

Full Title:

A methodology and a theoretical taxonomy for centrality measures: What are the best centrality indicators for student networks?

Short Title:

What are the best centrality indicators for student networks?

Kristel Vignery [1]¶*, Wim Laurier[1]¶

[1] Department of Economy & Management, Université Saint-Louis ■ Bruxelles, 1000 Brussels, Belgium.

* kristel.vignery@gmail.com; kristel.vignery@usaintlouis.be

¶These authors contributed equally to this work.

Acknowledgements

This research was made possible with the assistance of the University Saint-Louis - Brussels, and the authors would like to thank five faculty members in particular (Philippe Desmette, Mauricio Garcia, Gilles Grandjean, Alexandre Girard & Laurent Van Eynde) for facilitating data collection. The authors would also like to thank Professors François Fouss, Frédéric Nils & Marco Saerens for their valuable re-lecture, comments and suggestions.

Abstract

In social network analysis (SNA) several measures were developed to assess a nodes' centrality in a graph. Among these measures, we find connectivity or degree-based measures, geodesic distance-based indices, geodesic path-based scores and centrality measures related to a node's neighborhood. Moreover, studies that investigate networks use mostly degree, closeness and/or betweenness centralities, even if other measures might be valid candidates for representing and understanding these networks. The main contribution of this paper is the development of a methodology that allows comparing and validating centrality indices when studying a particular network of interest. The proposed methodology integrates the following steps: the choice of the centrality measures regarding the network of interest; the development of a theoretical taxonomy of these measures; the identification, by means of Principal Component Analysis (PCA), of latent dimensions of centrality within the network of interest; the verification of the proposed taxonomy of centrality measures; and the identification of the centrality measures that best represent the network of interest. Subsequently, we applied the proposed methodology on an existing graph of interest, i.e., on a real student network. We chose eighteen centrality measures that were developed in SNA, we thoroughly defined them, and we proposed a theoretical taxonomy of these eighteen measures. The PCA shows the emergence of six latent dimensions of centrality within the student network and, most importantly, shows that most of the centrality indices saturate on the same categories as those proposed by the theoretical taxonomy. Additionally, the results suggest that other indices than the most frequently applied might be more relevant for research on student networks.

Key words

Student networks, centrality dimensions, taxonomy, comparison of indices, selection of indices.

I. Introduction

Many centrality measures exist in social network analysis (SNA) for representing nodes within graphs. First, it is crucial to classify those centrality measures (i.e., to create taxonomies of centrality indices), and to verify such classifications by means of thorough methodologies applied on real data. Second, concerning real networks studies, in addition to the measures that are mostly used (i.e., degree, closeness, and betweenness centralities), alternative centrality indices could be considered when investigating such networks. Moreover, the appropriateness of the centrality measures - i.e., according to the nature of the studied graph - should also be taken into account in research on networks.

1.1. The relevance of centrality measures in networks

A network is a set of connected nodes (e.g., students within their peer group). In SNA, a major issue concerns how the nodes are characterized within a network. The literature recognizes the relevance of centrality for representing and characterizing nodes. The centrality concept is one of the most powerful indicators to represent a node because it gives information about its importance, prestige, prominence or involvement, about how a node get access to and spreads information, and about the node's proximity to phenomena that are observed within a network (Wasserman & Faust, 1994; Hanneman & Riddle, 2005; Kolaczyk & Gábor, 2014; Fouss & al., 2016). Various studies have used centrality measures to represent a network's nodes and eventually to represent the links between the nodes' centrality and variables of interest. Studies in management and organizational contexts, for instance, have used centrality measures to investigate job performance (e.g., Mehra & al., 2001; Sparrowe & al., 2001; Cross & Cummings, 2004; Zhang & Venkatesh, 2013), firm innovativeness and/or performance (e.g., Ahuja, 2000; Tsai, 2001), employee creativity (e.g., Perry-Smith, 2006), access to leadership jobs (e.g., Dhand & al., 2014) and information subsystems in manufacturing firms (Song & al., 2015). Studies in economy and finance have used centrality measures to explore research

domains such as the labor market (e.g., Montgomery; 1991; Calvo-Armengo & Jackson, 2004), inequalities (e.g., Neal, 2008; Clauset & al., 2014), productivity (e.g., Ductor & al., 2014) and liquidity flows (e.g., Bech & al., 2010). Marketing research has used centrality measures to look at customer retention behavior (e.g., Benoit & Van den Poel, 2012), consumer influence (e.g., Lee & al., 2010), brand switching behavior (e.g., Iacobucci & al., 1996), the processes behind adoption decisions (e.g., Katona & al., 2011), the spreading of viral campaigns (e.g., Kiss & Bichler, 2008; Zhang & al., 2010) and product network analysis (e.g., Kim & al., 2012). In sociology and political sciences, studies have used centrality measures to cover research topics such as national security, organized crime and terrorism (e.g., Krebs, 2002; Chen & al., 2004; Mastrobuoni & Patacchini; 2012; Alzaabi & al., 2015), elections and voting behavior (e.g., Xu & al., 2014; Burgess & Bruns, 2012) and policy networks including the assessment of key actors (e.g., Krackhardt, 1990; Lewis, 2006). Studies concerning biological networks have used centrality measures for topological analysis and the visualization of sub-communities emerging within a graph (e.g., Joy & al., 2005; Yu & al., 2007; Koschützki & Schreiber, 2008; Doncheva & al., 2012) or for the identification and modeling of central genes or proteins (e.g., Idowu & al., 2004; Zhang & Lin, 2008; Wang & al., 2011; Peng & al., 2015; Ashtiani & al., 2018).

1.2. The need for a taxonomy of centrality measures

Many centrality measures (e.g., degree, closeness, betweenness, eccentricity, geodesic k-path distance, eigenvector and page rank scores) were developed in SNA to assess node centrality within a graph (Kiss & Bichler, 2008; Landherr & al., 2010; Batool & Niazi, 2014; Song & al., 2015; Ghazzali & Ouellet, 2017; Ashtiani & al., 2018). However, literature reviews on centrality measures are rare (Lü & al., 2016). One of the objectives of this paper is to thoroughly define a number of centrality measures found in the literature. Then, according to Lü & al. (2016), it is important to compare and classify well-known centrality measures in order to highlight their similarities and differences. Another purpose concerns the comparison and the theoretical classification of centrality indices according to several criteria such as their formula, the benefits brought by high levels of centrality

(e.g., access to information), and the consideration (or not) of neighborhood properties such as prestige (e.g., Song & al., 2015; Lü & al., 2016; Ghazzali & Ouellet, 2017; Ashtiani & al., 2018). We have observed that such taxonomies of centrality measures are rare in the literature. Therefore, this paper proposes a theoretical classification of several centrality indices.

1.3. The need for a validation of taxonomies

In the literature, we found that the rare taxonomies of centrality measures were not systematically verified (e.g., on real data and/or by thorough methodologies). As taxonomies are a kind of ontology, which is a concept used in knowledge engineering and has been defined as '*a formal specification of a shared conceptualisation*' (Borst, 1997), their appeal to a community (i.e. sharedness) and their fit with the reality they represent (i.e. conceptualisation) need to be validated (Guarino & al., 2009). As such, they need to be sound, complete, lucid and laconic (Guizzardi, 2007). An ontology is sound when there is no construct excess and each of its constructs match an underlying reality in the intended universe of discourse (e.g. a student network). For example, a *sound* taxonomy of centrality measures for student networks only contains theoretical categories that faithfully represent a centrality notion or dimension for student networks in the real world. A *complete* ontology has no construct deficit and hence, a construct for each aspect of the underlying reality. For example, by means of an exhaustive list of theoretical categories, a complete taxonomy of centrality measures would reflect each centrality dimension existing within student networks (i.e. including those that have not been observed yet). A *lucid* ontology has no construct overload (i.e. homonymy) and hence only constructs that map to (at most) a single aspect of the underlying reality. For example, a lucid taxonomy would not contain a theoretical category that would refer to several latent dimensions of student centrality. A *laconic* ontology has no construct redundancy (i.e. synonymy) and hence, at most one construct for each aspect of the underlying reality. For example, a laconic taxonomy would contain only a theoretical category for each centrality dimension existing within student networks. In absence of these criteria, the taxonomy could lead to ambiguous interpretations of the centrality measures pertaining, for

instance, a student network. Therefore, this paper has the ambition to develop a methodology that verifies the proposed theoretical taxonomy on real data - i.e., a student network.

1.4. Student networks: the centrality question

Links between student centrality within their peer network and education outcomes are the object of many studies. Those researches concern performance and achievement (e.g., Thomas, 2000; Yang and Tang, 2003; Russo & Koesten, 2005; Cho & al., 2007; Zhang & al., 2008; Obadi & al., 2010; Hommes & al., 2012; Woolf & al., 2012; Gašević & al., 2013; Vaughan & al., 2015; de-Marcos & al., 2016; Mushtaq & al., 2016; Poldin & al., 2016; Zwolak & al., 2017; Liu & al., 2018; Saqr & al., 2018a; Saqr & al., 2018b; Vargas & al., 2018), but also learning (i.e., attitudes about the courses, sharing and construction of knowledge) (e.g., Russo & Koesten, 2005; De Laat & al., 2007), delinquency (i.e., petty crime and substance use behaviors) (e.g., Baerveldt & al., 2004; Cruz & al., 2012), sense of community (e.g., Ennett & al., 2006; Dawson, 2008; Shen & al.,2008) or dropout (e.g., Bayer & al., 2012; Yang & al., 2013). As observed in literature on other types of networks (e.g. biological networks in Ashtiani & al., 2018), the studies dedicated to student networks mostly conceptualized centrality as (1) the simplest measure of centrality - i.e., the degree centrality or number of entertained ties, (2) the closeness centrality - i.e., the distance with other nodes in the network - and/or (3) the betweenness index- i.e., the number of times that a node is located on shortest paths linking other nodes. Among these studies, some results showed positives effects of student centrality on education outcomes while others demonstrated none or even negative impacts. For instance, in regards with student performance, while the degree and closeness centralities seemed to have a positive effect on achievement, the impact of betweenness centrality appeared to be less clear.

The choice of the centrality measures might explain the nature of the observed links between centrality and education outcomes, along with the inconsistent results (Ashtiani & al., 2018). The way centrality is mathematically computed defines centrality and establishes how individuals are represented within a network. Moreover, studies showed that the ranks of the scores obtained for

different centrality indices do not always match (e.g., a node has high scores on some centrality measures, but average or low scores on other indicators of centrality) (Kiss & Bichler, 2008; Landherr & al., 2010). In order to identify central actors within a network, it is therefore crucial to test several centrality measures (i.e., based on the specific type of network and based on the centrality definitions), and to identify the most appropriate indices among those measures (Kiss & Bichler, 2008; Landherr & al., 2010; Batool & Niazi, 2014). Finally, as stated earlier, many other metrics than degree, closeness and betweenness were developed in SNA in order to assess a node's centrality within a graph. However, student networks were rarely represented using those alternative centrality measures. One additional objective of this paper is to highlight which centrality measures might be the most informative for student networks. This question has received little attention in student networks research, and the literature on the topic mainly concerns other types of networks than student graphs (e.g., biological networks, terrorist cells, customer networks). As Ashtiani & al. (2018) argue for biological networks, we argue there is a need for guidelines pertaining the relevance of centrality measures for student networks. This work aims at selecting the best suited centrality measures for representing a student network or for studying the impacts of student networks on education outcomes such as student performance. Using relevant centrality indices might enable a deeper understanding of student networks and of the mechanisms within those networks.

1.5. Scope of the research and organization of the paper

The paper's main contribution is the proposal of an integrated methodology that allows for choosing, comparing and verifying centrality indices. A subsequent contribution is the application of the proposed methodology on an existing graph of interest, i.e., a real student network. The application of the methodology on a real student network allows to:

(1) Thoroughly chose a set of centrality measures that are relevant for student networks and thoroughly define these measures,

(2) Propose a theoretical taxonomy of those centrality measures based on criteria such as their definition, logic and formula,

(3) Verify the proposed taxonomy, by means of latent centrality dimensions that are highlighted within a real student network,

(4) Among the chosen set, identify the centrality measures that are the most representative and significant for representing a student network.

To the best of our knowledge, the development of such methodology, together with its application on real data, have never been undertaken.

The second section of the paper presents the integrated methodology. The third section presents the data and the student network drawn from the data. The fourth section presents the results of the study: one the one hand the comparison between the latent dimensions of centrality resulting from the PCA procedure and the theoretical classification of the centrality measures, and on the other hand the centrality measures that contribute the most to representing the student network. Finally, the last section discusses the results, the limitations of this study and points out the need for further research.

II. The proposal of an integrated methodology

Fig 1 shows the integrated methodology. The first step consists of choosing a set of centrality indices according to the nature of the considered graph (e.g., undirected versus directed) but also according to the definition of the centrality indices, which must be evaluated in the light of the network's type (social, biological, financial …) and in the light of the research questions. Second, a theoretical taxonomy of centrality measures is proposed. Third, centrality indices are computed on a real network. Fourth, a PCA is applied on the computed centrality measures, and its outputs are used in order (1) to verify the theoretical taxonomy of centrality indices by comparing this theoretical classification to factorial components emerging from the PCA, and (2) to determine the most informative centrality measures for the considered network.

Fig 1. Choosing, comparing and verifying centrality indices on networks: An integrated methodology

2.1. Choosing and defining the centrality measures

We obtained a list of suitable centralities based on our student network's topology by using the package CINNA (Ashtiani & Jafari, 2017) implemented in R©, and more precisely the function *proper_centralities*. The function output – i.e., the complete list of the suitable centralities applicable to our student network - is presented in Appendix 1. Among those suitable centralities, the measures for representing our student network were selected by means of the sequence shown in fig 2 and composed of the following steps:

Fig 2. Choosing networks' centrality indices: sequence

(1) *Who* are the nodes, *what* is the network's type? (i.e., in our case, a directed graph of students);

(2) Which type of centrality might be suitable to investigate when we study the links between student centrality and educational outcomes such as learning, academic performance, dropout. Indices considered as irrelevant in regard to our network and to research questions related to the network were not selected for further analyses;

(3) For the remaining indices: we analyzed if there were any measures whose formula was highly similar (i.e., that differ only by very few parameters). For instance, the communicability betweenness centrality, the flow betweenness centrality, the load centrality and the stress centrality are each variants of the betweenness centrality. In those cases, we chose the centrality index figuring in the highest number of documents on Google Scholar (e.g., for the betweenness centrality, the measure that was proposed by Freeman in 1979);

(4) For the remaining indices: in order to continue the methodological process with a reasonable number of indices, centrality measures that figured in very few documents on Google Scholar were not selected for further analyses.

Among the complete list of suitable centralities presented in Appendix 1, the set of the chosen centrality measures is composed of the eighteen following indices. We consider a centrality measure that takes into account the edges direction (i.e., that can be computed separately on the incoming and on the outgoing ties) as two distinct indices:

- Eccentricity centrality (computed separately on the incoming and the outgoing ties)
- Closeness centrality (computed separately on the incoming and the outgoing ties)
- Residual closeness centrality (computed separately on the incoming and the outgoing ties)
- Betweenness centrality
- Geodesic k-path centrality (computed separately on the incoming and the outgoing ties)
- Bottleneck centrality (computed separately on the incoming and the outgoing ties)
- Eigenvector centrality
- Hub score
- Authority score
- Pagerank score
- MNC - maximum neighborhood component (computed separately on the incoming and the outgoing ties)
- Cross-clique connectivity

Then, a deep understanding of these measures is a necessary condition for, one the one hand, pursuing the different objectives of the methodology, and on the other hand, enabling deeper knowledge of student networks and of the mechanisms occurring within those networks. Thorough definitions of these eighteen centrality measures are given in Appendix 2.

2.2. Proposal of a theoretical taxonomy

Based on examples of taxonomies from Song & al. (2015), Lü & al. (2016); Ghazzali & Ouellet (2017) and Ashtiani & al. (2018), who each worked on different sets of centrality measures, we

propose five theoretical categories to classify the eighteen centrality measures (see table 1). As the definitions of the eighteen indices show (see Appendix 2), the first category - i.e., distance-based - assesses the proximity of a node with the other members of the graph. The second category - i.e., geodesic path-based - is related to the geodesic paths in which nodes are located. The third category is based on the connectivity or degrees, i.e., on the number of direct connections a node possesses. Finally, the fourth and five categories take the neighborhood of a node into account, the former the neighbors' prestige and the later the topology of the members adjacent to the node under consideration.

2.3. Computation of the centrality measures on a real network

We used the igraph package (Csardi & Nepusz, 2006) implemented in R© to compute the eighteen centrality measures on our student network.

Table 1. Centrality measures taxonomy: categories.

	Topological structure of the network			Neighborhood	
Category number	1	2	3	4	5
Logic & formula	Geodesic distance based	Geodesic path based	Connectivity based	Prestige of Neighborhood	Topology properties of neighborhood
Process(es) related to the centrality	(Speed of) access to information	Control over the information<br><br>Diffusion of Information<br><br>Bridge role	Power and influence	Power and influence	Diffusion of Information<br><br>Cohesiveness role
Eccentricity *(in- & out-)*	●				
Closeness *(in- & out-)*	●				
Residual closeness *(in- & out-)*	●	●			
Geodesic k-path *(in- & out-)*	●	●			
Betweenness		●			
Bottleneck *(in- & out-)*		●			
Eigenvector prestige score			●	●	
Hub & Authority scores			●	●	
Page rank			●	●	
Cross-clique connectivity			●		●
MNC *(in- & out-)*					●

2.4. The PCA as methodological tool

The PCA is a factorial analysis method which uses the correlations (i.e., inter-dependencies) between variables - in our case the centrality indices - to reduce the p dimensional space of these variables into a k dimensional space (with $k < p$). PCA results in a minimal number of principal components (i.e., factorial axis or latent dimensions) that corresponds to maximum data dispersion, these principal components being linear combinations of the initial variables. First, we performed PCA on our centrality indices to highlight the k latent dimensions of centrality within our student network. Also, by computing the coordinates of each variable on each highlighted dimension, PCA enables for identifying the factor on which a variable saturates the most, which allows for identifying the centrality indices that belong to the same latent dimension. The comparison of the PCA output - the k highlighted latent dimensions - with the proposed theoretical classification of centrality indices then allowed for verifying whether or not this theoretical classification could be validated. Second, we performed PCA to determine the most representative centrality measures (among the complete set of the eighteen measures) for student networks (Ashtiani & al., 2018).

We worked on standardized data to neutralize the problem of variables with different units. We used the *Varimax* procedure that ensures a better repartition of the variables on the factors by rotating the axis, and who allows an easier interpretation of the factorial axis (Hair & al., 2010; Tufféry, 2012).

III. Data

The data were collected in October 2016 at Saint-Louis University in Brussels, Belgium. 574 first-generation freshmen students (i.e., students registered in their first year of studies and for the first time) were interrogated about their friendship ties at university. In the survey, the student' friends were described as the '*persons with whom students spend personal time, with whom they interact on a regular basis (in face to face, by phone or on online social medias), that they see outside classes, that they trust, and/or with whom they share their personal issues*' (Thomas, 2000; Cho & al., 2007;

Hommes & al., 2012).The nodes graph, i.e., the student network, was drawn from the collected data. Since the survey was not mandatory, students who did not participate could nevertheless be cited as ties - a case of missing or non-respondent actors (Robin & al., 2004). A thorough analysis of our graph reveals that 296 students were nominated at least one time by the 574 respondents but did not complete the survey. According to Wasserman & Faust (1994), SNA methods require the complete recording of interactions between actors belonging to the studied network. Using the *respondent only approach* (i.e., in our case deleting the nominations that correspond to the 296 students who did not participate) instead of working with the *all cases approach* (i.e., in our case including the 296 missing actors in further analyses), can bias the indices (e.g., the centrality measures) that are computed on the graph (Gile & Handcock, 2006; Huisman, 2009; Gile & Handcock, 2017). We used the Exponential Random Graph Models (ERGMs) (Robin & al., 2004) to impute friendship relations for the 296 missing actors by means of statnet (Handcock & al., 2008) with the ergm package (Hunter & al., 2008) implemented in R©. This imputation allowed for computing centrality measures for each of the 870 students belonging to the augmented network (i.e., the 574 respondents and the 296 missing actors for which ties were imputed).

IV. Results

4.1. Correlations between the centrality measures

We computed the eighteen centrality measures for each node (i.e., for each student). The ρ correlation coefficients between each centrality measure together with their significance levels are showed in Table 2. First, results show that the correlations between centrality measures that can be computed separately on the incoming and the outgoing ties (e.g., the eccentricity *in*- and *out*-centralities) are all positive and significant (*p-values* ≤ 0.05). Second, results show many positive and significant correlations between centrality measures (*p-values* ≤ 0.05), except for the bottleneck indices (*in*- and *out*-), for which few significant correlations are observed. We notice the strongest relationships between: the eccentricity and closeness *in*- centralities ($\rho = 0.96$); the eccentricity and

closeness *out*- centralities (ρ = 0.97); the residual closeness and geodesic k-path *in*- centralities (ρ = 0.97); the residual closeness and geodesic k-path *out*- centralities (ρ = 0.97); the betweenness with the residual closeness *in*- (ρ = 0.62), the geodesic k-path *in*- (ρ = 0.62) and the geodesic k-path *out*- (ρ = 0.62) centralities; the eigenvector prestige and the Kleinberg's authority centrality scores (ρ = 0.97); the Kleinberg's hub centrality with the Kleinberg's authority centrality (ρ = 0.86) and the eigenvector prestige scores (ρ = 0.85); the Page rank score with the residual closeness (ρ = 0.51) and the geodesic k-path (ρ = 0.49) *in*- centralities; the cross-clique connectivity with the maximum neighborhood component (*in*- : ρ = 0.78, and *out*- : ρ = 0.72).

Table 2. Correlations between the eighteen centrality measure.

	Ecc *in-*	Ecc *out-*	Clos *in-*	Clos *out-*	Res clos *in-*	Res clos *out-*	Between	k-path *in-*	k-path *out-*	Bottle *in-*	Bottle *out-*	Eigen.	Page rank	Authority	Hub	MNC *in-*	MNC *out-*	Clique
Ecc *in-*	1,00																	
Ecc *out-*	**,17**	1,00																
Clos *in-*	**,96**	**,18**	1,00															
Clos *out-*	**,15**	**,97**	**,18**	1,00														
Res clos *in-*	**,44**	**,23**	**,63**	**,26**	1,00													
Res clos *out-*	**,13**	**,59**	**,19**	**,72**	**,36**	1,00												
Between	**,18**	**,32**	**,29**	**,40**	**,60**	**,62**	1,00											
k-path *in-*	**,33**	**,21**	**,51**	**,24**	**,97**	**,35**	**,62**	1,00										
k-path *out-*	**,12**	**,46**	**,17**	**,60**	**,33**	**,97**	**,62**	**,34**	1,00									
Bottle *in-*	-,01	-,04	,00	-,03	-,02	,02	-,01	-,02	,03	1,00								
Bottle *out-*	,01	-,02	,01	-,01	,03	,00	,04	,04	-,01	**,21**	1,00							
Eigenvector	,00	,02	**,08**	**,06**	**,21**	**,10**	**,09**	**,21**	**,10**	,00	**,06**	1,00						
Page rank	**,26**	**-,07**	**,35**	**-,10**	**,51**	-,03	**,26**	**,49**	-,03	,02	**,07**	,08	1,00					
Authority	,01	,02	**,08**	**,07**	**,20**	**,12**	**,11**	**,21**	**,13**	-,02	,05	**,97**	**,06**	1,00				
Hub	,00	,04	**,06**	**,10**	**,16**	**,18**	**,10**	**,16**	**,18**	,00	**,06**	**,85**	**,06**	**,86**	1,00			
MNC *in-*	**,27**	**,21**	**,35**	**,25**	**,50**	**,33**	**,32**	**,48**	**,31**	,03	**,08**	**,35**	**,30**	**,37**	**,31**	1,00		
MNC *out-*	**,13**	**,37**	**,20**	**,41**	**,32**	**,48**	**,24**	**,31**	**,44**	**,10**	**,08**	**,32**	**,24**	**,34**	**,43**	**,68**	1,00	
Clique	**,15**	**,25**	**,25**	**,33**	**,49**	**,51**	**,43**	**,50**	**,50**	,02	**,10**	**,43**	**,22**	**,46**	**,48**	**,78**	**,72**	1,00

In bold front: coefficients for which the p-value is ≤ 0.

4.2. The centrality latent dimensions within student networks and the verification of the taxonomy

Three conditions are necessary for PCA to be relevant. First, the variables must be correlated, which seems to be the case as shown above. Second, the Bartlett's test verifies whether highly correlated variables might be correlated to the same latent factor(s) (Malhotra & al., 2007; Hair & al., 2010). Being significant (*p-value* = 0.000), the PCA seemed relevant. Third, the index of Kaiser-Meyer-Olkin (KMO) tests the compressibility of information. The value of the *KMO* (= 0.713) being higher than 0.5 - the critical threshold - we can consider the factorization as statistically acceptable.

Concerning the quality of a variable's representation, 50% of the information contained in each variable must be preserved in the factorial plan (Hair & al., 2010), which was the case for all our eighteen centrality measures. Then, to determine the minimum number of axis that preserves the maximum percentage of information, we used the Kaiser criterion that consist of keeping the *k* factorial axes possessing eigenvalues (i.e. projected inertia or information's degree preserved by an axis) that are higher than 1 (Malhotra & al., 2007; Hair & al., 2010). According to this criterion, six latent dimensions were retained. 82.82% of the total inertia present in the original dimensional space is conserved into these six components (see table 3). This respects an additional criterion based on a threshold of 60% for the minimum percentage of conserved variance in the factorial plan (Malhotra & al., 2007; Hair & al., 2010).

Table 3. Variance explained by the six firsts factorial axis.

	Initial Eigenvalues		
Components	Total	% of Variance	Cumulative %
1	6,07	33,71	33,71
2	2,80	15,54	49,25
3	2,48	13,77	63,02
4	1,32	7,33	70,35
5	1,23	6,82	77,17
6	1,02	5,65	82,82

Table 4 shows the variables and their factor loading on the component for which their saturation is the highest. The closeness, residual closeness, eccentricity and geodesic k-path *out*- centralities are

correlated with the first factorial axis. According to the definitions and formula (in Appendix 2) of these four indices, this first component or latent dimension might therefore reflect the ease with which a node reaches the other nodes, connects them and transmits information throughout the network. The second dimension, highly correlated with the Kleinberg's authority & hub centrality scores and with the eigenvector prestige score, relates to centrality through the number of connections with prestigious friends. The geodesic k-path and residual closeness *in*- centralities, the betweenness and the Page rank score load on the third factor. This dimension might therefore denote the ability to control the received information, and a node's degree of significance, especially by being located on the (local) shortest paths converging toward the node. The fourth dimension (the maximum neighborhood components and the cross-clique connectivity) is linked to the degree of cross-connectivity of a student and of its neighbors. The fifth component, highly correlated with the eccentricity and closeness *in*- centralities, relates to the ease with which a node is reached by the other nodes in the network and to its ability to receive information. The sixth and last dimension reflects the degree of bottleneck, i.e., the degree of confluence through a given student.

Table 4. Results of the Varimax rotation: coordinates of each variable on the factorial axis on which the saturation is the highest.

	Components					
Centrality Indices	1	2	3	4	5	6
Closeness centrality (*out*-)	.91					
Residual closeness centrality (*out*-)	.88					
Eccentricity centrality (*out*-)	.83					
Geodesic k-path centrality (*out*-)	.81					
Kleinberg's authority centrality scores		.96				
Eigenvector prestige score		.96				
Kleinberg's hub centrality scores		.91				
Geodesic k-path Centrality (*in*-)			.86			
Residual closeness centrality (*in*-)			.83			
Betweenness			.74			
Page rank			.60			
MNC - maximum neighborhood component (*out*-)				.86		
MNC - maximum neighborhood component (*in*-)				.82		
Cross-clique connectivity				.72		
Eccentricity centrality (*in*-)					.94	
Closeness centrality (*in*-)					.91	
Bottleneck centrality (*in*-)						.79
Bottleneck centrality (*out*-)						.76

In order to verify the proposed taxonomy, we compared the theoretical classification (in Table 1) with the six centrality dimensions emerged from the PCA - i.e., with their composition in terms of the eighteen centrality indices (in Table 4).

Within the taxonomy, four indices are gathered within a first category (i.e., the category number 1 in Table 1), which is built on the criterion of a geodesic distance-based formula and on the criterion of access to information as centrality corollary: the eccentricity, closeness, residual closeness and geodesic k-path centralities. Those four indices, but computed for the *outgoing* ties only, saturate on the first latent dimension in the PCA (Table 4), which therefore matches with the category number 1 in Table 1. This theoretical category is therefore validated, but only for centralities computed on the nominations that are made by a node. Moreover, the closeness and eccentricity centralities that are computed on the *incoming* ties - i.e., the nominations received by a node - and that both saturate on the fifth factorial axis (Table 4), seem to form a subset within the first theoretical category in Table 1. Then, in the theoretical taxonomy, we assigned the residual closeness and the geodesic k-path centralities to the first category, but also to a second family of indices (i.e., category number 2 in Table 1) that are based on a geodesic-path formula, and that relies on information control and diffusion. As seen above, residual closeness and geodesic k-path *out*- centralities have been verified as being part of the theoretical category number 1. However, they are validated for the theoretical category number 2 when they are computed on the incoming ties. They form a latent construct (i.e., the third dimension in Table 4) together with the betweenness index, the latter being also validated for the theoretical category number 2 of centrality measures. As shown for the eccentricity and the closeness centralities, according to the nature of the ties (i.e., *in*- versus *out*-), the residual closeness and geodesic k-path indices seem therefore to be divided into two distinct categories.

Then, the second latent dimension resulting from the PCA (Table 4), that gathers the Kleinberg's authority & hub centrality scores together with the eigenvector prestige score, validates the third and fourth theoretical categories of indices within the taxonomy (Table 1), i.e., the categories based respectively on the degree of connectivity and on the prestige of the connections, who both reflect power and influence.

The fifth theoretical category in Table 1 concerned centrality indices who take the topology properties of the neighborhood into account in their formula and who relate to information diffusion and to cohesiveness roles. The cross-clique connectivity and the maximum neighborhood components (*in*- and *out*-) were proposed as being part of this category, which is confirmed by the PCA, those three indices gathering on a same latent factor (i.e., the fourth dimension in Table 4). It should be noted that since a clique is composed of three or more nodes, the cross-clique connectivity was also proposed as part of the third theoretical category in Table 1, which is based on the number of connections. However, the PCA confirms that cross-clique connectivity belongs to the same category as the MNC scores.

Finally, bottleneck (*in*- and *out*-) and Page rank scores do not behave as expected according to the taxonomy. First, as shown above with the correlations and the PCA, the two bottleneck indices do not correlate significantly with most of the other sixteen centrality measures, and both saturate on a specific factorial axis (i.e., the sixth dimension in Table 4). Yet based on shortest paths in their algorithm, bottlenecks seem therefore to measure a different type of centrality than the residual closeness, geodesic k-path and betweenness indices. Second, we expected the Page Rank score to be validated within the same theoretical category as the eigenvector and the Kleinberg's authority & hub scores, since the Page rank formula takes into account the prestige of the incoming ties when computing a node's centrality. Instead, PCA shows a maximum saturation of Page rank on the same factorial axis as the geodesic k-path (*in*-), the residual closeness (*in*-) and the betweenness centralities.

Table 5 summarizes the above : the first latent dimension validates the first theoretical category, but for indices computed on the outgoing ties only, while the fifth dimension matches, but only for incoming ties, with two centrality measures that were proposed within the theoretical category number 1. A unique dimension (i.e., the dimension number 2) validates the proximity of indices that were proposed as belonging to two theoretical categories (i.e., the third and fourth categories in Table 1). Then, except for the Page rank score (for which we expected a saturation on the second dimension) and except for the two Bottleneck indices (who both saturate on the sixth dimension), the third

dimension matches with the theoretical category number 2. Finally, the fifth theoretical category of centrality measures is validated by the fourth PCA dimension.

Table 5. Centrality dimensions emerged from the PCA and theoretical classification: Comparison.

Centrality measures	Latent dimensions (Table 4)	Taxonomy categories (Table 1)
Eccentricity *(out-)*<br>Closeness *(out-)*<br>Residual closeness *(out-)*<br>Geodesic k-path *(out-)*	1	1
Eigenvector prestige score<br>Hub score<br>Authority score	2	3 & 4
Page rank	3	3 & 4
Residual closeness *(in-)*<br>Geodesic k-path *(in-)*<br>Betweenness	3	2
Cross-clique connectivity<br>MNC *(in-)*<br>MNC *(out-)*	4	5
Eccentricity *(in-)*<br>Closeness *(in-)*	5	1
Bottleneck *(in-)*<br>Bottleneck *(out-)*	6	2

As detailed above, the first category is based on geodesic distance and access to information, the second category is based on geodesic path and diffusion of information, the third category is based on connectivity and power, the fourth category is based on neighborhood's prestige and on power, and the fifth category is based on neighborhood's topology and diffusion of information.

4.3. The most representative measures of centrality for student networks

One of the objectives of the paper was to find the best centrality measures - i.e., the most representative and significant indices - when we investigate and represent student networks. As in Ashtiani & al. (2018) for biological networks, our goal was therefore to establish, among a set of centrality indices, the measures that best categorize the central students from the peripherical ones.

First, we retrieved the relative contribution of each centrality index (i.e., each p variable) for each of the six dimensions (i.e., the k factorial axes) that were retained in the PCA. The following formula computes a variable's contribution for a factorial axis:

$$\frac{\rho\left(X_p, F_k\right)^2}{\lambda_k} \qquad (1)$$

Where $\rho\left(X_p, F_k\right)^2$ represents the quality of the variable's representation on the factorial axis k, and is equal to the square correlation coefficient between the variable X_p and the axis F_k; and λ_k represents the variance or inertia preserved on the factorial axis k, and is equal to the sum of the square correlation coefficients between each variable and k (Tufféry, 2012).

Then, we computed the average contribution of each centrality measure to the factorial plan (i.e., the average contribution on the six factorial axes). We compared each average contribution to a threshold of $(1/18) \times 100 = 5.55\%$, i.e., a centrality measure's theoretical contribution since there are eighteen indices. Higher (resp. lower) values than 5.55% indicate a contribution that is above (resp. below) the theoretical average contribution. Table 6 shows the centrality measures and their average contribution (by descending order) to the factorial plan. The eight indices that best represent the variance of student centrality within a network are the bottleneck indices (*in*- & *out*-), the eccentricity and closeness centralities computed on the incoming ties, the maximum neighborhood component measures (*in*- & *out*-) and the Kleinberg's authority & eigenvector prestige scores. On the contrary, other centralities (e.g., the betweenness index and the eccentricity and closeness centralities computed on the outgoing ties) seem to contribute less to the factorial plan, being below the average threshold of 5.55%.

Table 6. Average contributions of the centralities on the factorial plan.

	Average contribution to the factorial plan (Pct.)
Bottleneck (in-)	**8,50**
Bottleneck (out-)	**7,97**
Eccentricity (in-)	**7,95**
Closeness (in-)	**6,77**
MNC (out-)	**6,38**
MNC (in-)	**5,80**
Kleinberg's authority scores	**5,67**
Eigenvector prestige score	**5,64**
Betweenness	5,53
Kleinberg's hub scores	4,94
Closeness (out-)	4,76
Eccentricity (out-)	4,63
Geodesic k-path (in-)	4,63
Page rank	4,22
Residual closeness (in-)	4,21
Cross-clique connectivity	4,11
Residual closeness (out-)	3,89
Geodesic k-path (out-)	3,69

V. Discussion

We applied the integrated methodology that we developed - i.e., (1) the choice, definition and proposal of a theoretical classification of centrality measures, (2) the highlighting of centrality dimensions within a network of interest, (3) the verification of the proposed theoretical taxonomy by means of those dimensions, and (4) the identification of representative centrality indices - on a real student network.

In accordance with other studies conducted on other types of networks (e.g., sociometric networks in Valente & al., 2008; social, ecological and neural networks in Batool & Niazi, 2014; terrorist and viral networks in Ghazzali & Ouellet, 2017), our results show positive and significant correlations between several centrality measures (e.g., between the eccentricity and the closeness centralities, between the geodesic k-path centrality and the betweenness index, between the closeness centrality and the eigenvector prestige score…). Concerning the centrality dimensions that exist within student networks, our results show the emergence of six latent constructs: (1) the ability for a student to reach and to transfer information, (2) its ability to be reached and to receive information, (3) its significance for the network structure and its control over the information flow, (4) its importance through the number of connections with prestigious students, (5) its degree of cross-connectivity, and (6) its position as a confluent node. First, these results indicate that a centrality measure that is computed for the incoming links seems to differ from the same centrality measure that is computed for the outgoing ties in its meaning and in its impacts on nodes. Our results show that the eccentricity, closeness, residual closeness and geodesic k-path centralities that are computed for the outgoing ties saturate on a different latent construct than the eccentricity and the closeness centralities that are computed for the incoming links. In regard to student networks, this result implies that it is not because a student is close to the other nodes of the network through its outgoing ties that he or she is automatically close to the other nodes of the network through its incoming connections. This also demonstrates that in student networks, the access to information might be different depending on a node's incoming and outgoing ties. Then, according to the nature of the ties (i.e., *in*- versus *out*-), the

residual closeness and geodesic k-path centralities are also divided into two categories or dimensions. In other words, the number of nodes that a student can reach - the student being located on (local) geodesic paths - might differ from the number of nodes that can reach the student, also through (local) geodesic paths. Moreover, the fact that residual closeness and geodesic k-path centralities are divided into two dimensions shows that the outgoing links seem important for the access to information, while the incoming ties appear relevant for information control. In conclusion, a node might be highlighted as significant when centralities are computed on its incoming (resp. outgoing) links, but not shown as central when its outgoing (resp. incoming) ties are used in the computations. Second, PCA shows that Page rank saturates on the same factorial axis as the geodesic k-path (*in*-), the residual closeness (*in*-) and the betweenness centralities. As far as student networks are concerned, these results suggest that students highlighted as central, because they are cited by many other students having a high degree of Page rank, are also significant through a high number of neighbors that can reach them (i.e., those neighbors being located at maximum *k* steps towards them). On this basis, we might infer that students having a high Page rank score are geographically close to the student(s) they cite, and belong to the same neighborhood as these students' closest neighbors.

Related to the validation of the theoretical taxonomy, except for Bottleneck and Page Rank scores, the five proposed categories of the theoretical classification are for the most part validated, since they match the latent dimensions highlighted by the PCA. The integrated methodology applied on real data however allowed improving the taxonomy by adding some granularity, for instance by showing that the direction of the ties should be considered in a theoretical classification of centrality measures. Specifically, regarding the four necessary criteria of taxonomies (i.e., sound, complete, lucid and laconic), the methodology, tested on real data, showed that:

- The proposed taxonomy seems to be sound (i.e., does not contain useless constructs) since each proposed theoretical category of indices matches with one latent dimension of centrality within the real network.

- In order to be complete (i.e., to cover each aspect of the centrality notion within a student network), an additional category within the theoretical taxonomy should be proposed for the Bottleneck centrality, which seems to cover a particular type of centrality.
- In order to be to be lucid (i.e. containing categories that map to (at most) a single aspect of centrality), as explained above, categories that take the direction of the ties into account should be added to the taxonomy.
- In order to be laconic (i.e., with no construct redundancy), the two categories *'Connectivity based*' (i.e., category number 3 in Table 1) and *'Prestige of Neighborhood*' (i.e., category number 4 in Table 1) should be merged into a single construct, since they do not seem to relate to different aspects of centrality (i.e., since the indices proposed for both categories - i.e., the eigenvector prestige score and the Kleinberg's authority centrality scores - saturate on only one latent dimension).

Concerning the centrality measures that should be chosen to describe and represent students within their network, as in Batool & Niazi (2014) and in Ashtiani & al. (2018), our results show centrality indices that contribute the most to the construction of the factorial plan and that best reflect the variability of centrality within student networks. For instance, future studies could use these four measures that were rarely or not yet used in the literature on student networks: (1) the bottleneck index computed on ingoing ties, (2) the eccentricity *in*- centrality, (3) the maximum neighborhood component measure (*out*-), and (4) the Kleinberg's authority score. Using these indices is expected to allow for capturing and investigating different dimensions of student centrality (i.e., its degree of confluence, its ability to be reached and to receive information, its degree of cross-connectivity and its centrality through prestigious connections), while making sure to select the best centrality candidates - i.e., which reflect a maximum of variability between students.

Two limitations of this research must be pointed out. First, as explained above, 50% of the information contained in a variable must be preserved in the factorial plan in order to consider its representation as sufficient and of good quality. Each variable met this requirement, but in comparison

with the other centrality measures (for which the representation was above or equal to 78%), the Page rank score and the bottleneck indices presented lower percentages of contained information (59% for the Page rank score, 59% for the bottleneck computed on the outgoing ties and 63% for the bottleneck computed on the incoming ties). If a variable is not well represented in the factorial plan, its proximity with other(s) variable(s) on the factorial plan may be misinterpreted (i.e., proximity may be assimilated as correlation whereas it is not the case in reality) (Tufféry, 2012). In our case, while the page rank score has a percentage of contained information that is above than 50% - the critical threshold – its proximity with the geodesic k-path Centrality (*in*-), with the residual closeness centrality (*in*-) and with the betweenness should be considered with caution. The considered proximity between the two bottleneck's scores should also be validated by subsequent studies. The second limitation relates to the high proportion (i.e., 34%) of missing actors or non-respondents (i.e., students that were cited by the respondents, but that did not complete the survey). Several authors (e.g., Huisman, 2009; Žnidaršič & al., 2012) showed that high levels of survey non-response impact the structural properties of social networks and might cause underestimation of the computed coefficients (Kossinets, 2006). Moreover, as explained above, the complete recording of interactions between actors belonging to the studied network is required in SNA. In order to fulfill this condition, two methodological approaches can be used: the *complete cases approach* or *respondent only approach* (i.e., deleting the nominations corresponding to students that do not complete the survey) or the *imputation approach* (i.e., imputing ties for the missing actors). Since the *respondent only approach* might produce more biased estimates than the *imputation approach* (e.g., Huisman, 2009; Wang & al., 2016; Gile & Handcock, 2017) we chose to impute ties for the non-respondents, by means of ERGMs, which give better performances than simpler imputation procedures (Huisman & al., 2018).

Three perspectives could be pursued in future research. First, the theoretical taxonomy could be tested on other student networks, but also within other contexts than educational settings - i.e., on other types of networks (e.g., organizational, biological …), in order to verify its validity (i.e., its soundness, lucidity and laconic nature) and to generalize its results. Also, in order to ensure its completeness and to include centrality measures that have not been observed yet, other measures than

those that were chosen in this paper should be tested. Second, our study should be replicated on other student networks in order to validate the best centrality measures when we investigate such graphs. Batool & Niazi (2014) emphasized the need to pursue research that identifies the best centrality measures for a given type of network (including the measures usually employed but also less traditional indices). Moreover, as stated by Landherr & al. (2010), Batool & Niazi (2014) and Ghazzali & Ouellet (2017), more studies that compare and formalize centrality measures within different contexts are necessary. Centrality measures considered as appropriate for a given network may not be able to correctly identify central nodes within other graph typologies (Ashtiani & al., 2018). The third perspective concerns frameworks or methodologies that could be used to verify the appropriateness of node centralities for different types of networks. Like Ashtiani & al. (2018), we argue that the choice and identification of the best centrality candidates should be the first step when investigating networks and when identifying its key players. The integrated methodology that we propose might therefore be useful for future studies related to networks and node centralities (i.e., to test any set of indices on any type of network).

VI. Conclusion

In this research, we proposed a theoretical taxonomy of a chosen set of centrality indices that we thoroughly described, we highlighted latent centrality dimensions that exist within student networks, we verified and we improved the proposed taxonomy on an existing student network, and we pointed out which centrality measures should be used when investigating student networks. We also presented an integrated methodology that allows pursuing these objectives and that might be applied to other centrality indices and/or on other types of networks. Our results demonstrate that for student networks, the direction of the ties (i.e., the incoming versus outgoing links) should be considered in the centrality computations, since they bring more information about a student's centrality within its peer network. Results also show that when dealing with student networks, centrality measures that best represent the six factorial axes that emerged from the PCA (i.e., the six indices that saturate the most on each factorial axis) should be integrated in future studies since they seem to cover different latent

dimensions of centrality. Finally, our results encourage using other indices than those usually employed when investigating student networks.

Acknowledgements

This research was made possible with the assistance of the University Saint-Louis - Brussels, and the authors would like to thank five faculty members in particular (Philippe Desmette, Mauricio Garcia, Gilles Grandjean, Alexandre Girard & Laurent Van Eynde) for facilitating data collection. The authors would also like to thank Professors François Fouss, Frédéric Nils & Marco Saerens for their valuable re-lecture, comments and suggestions.

References

[1] Ahuja, G. (2000). Collaboration networks, structural holes, and innovation: A longitudinal study. *Administrative science quarterly*, *45*(3), 425-455.

[2] Alahakoon, T., Tripathi, R., Kourtellis, N., Simha, R., & Iamnitchi, A. (2011). *K-path centrality: A new centrality measure in social networks*. In Proceedings of the 4th workshop on social network systems (pp 1-6).

[3] Alzaabi, M., Taha, K., & Martin, T. A. (2015). Cisri: A crime investigation system using the relative importance of information spreaders in networks depicting criminals communications. *IEEE Transactions on Information Forensics and Security*, *10*(10), 2196-2211.

[4] Asgari, Y., Salehzadeh-Yazdi, A., Schreiber, F., & Masoudi-Nejad, A. (2013). Controllability in cancer metabolic networks according to drug targets as driver nodes. *PloS one*, *8*(11), e79397.

[5] Ashtiani, M., Salehzadeh-Yazdi, A., Razaghi-Moghadam, Z., Hennig, H., Wolkenhauer, O., Mirzaie, M., & Jafari, M. (2018). A systematic survey of centrality measures for protein-protein interaction networks. *BMC systems biology*, *12*(1), 80.

[6] Ashtiani, M., & Jafari, M. (2017). CINNA: Deciphering Central Informative Nodes in Network Analysis.

[7] Aytac, A., & Berberler, Z. N. O. (2017). Residual closeness for helm and sunflower graphs. *TWMS Journal of Applied and Engineering Mathematics*, *7*(2), 209.

[8] Baerveldt, C., Van Rossem, R., Vermande, M., & Weerman, F. (2004). Students' delinquency and correlates with strong and weaker ties: A study of students' networks in Dutch high schools. *Connections*, *26*(1), 11-28.

[9] Batool, K., & Niazi, M. A. (2014). Towards a methodology for validation of centrality measures in complex networks. *PloS one*, *9*(4), e90283.

[10] Bayer, J., Bydzovská, H., Géryk, J., Obsivac, T., & Popelinsky, L. (2012). Predicting Drop-Out from Social Behaviour of Students. *International Educational Data Mining Society*.

[11] Bech, M. L., Chapman, J. T., & Garratt, R. J. (2010). Which bank is the "central" bank?. *Journal of monetary economics*, *57*(3), 352-363.

[12] Benoit, D. F., & Van den Poel, D. (2012). Improving customer retention in financial services using kinship network information. *Expert Systems with Applications*, *39*(13), 11435-11442.

[13] Bonacich, P. (1972). Factoring and weighting approaches to status scores and clique identification. *Journal of mathematical sociology*, *2*(1), 113-120.

[14] Bonacich, P. (1987). Power and centrality: A family of measures. *American journal of sociology*, *92*(5), 1170-1182.

[15] Bonacich, P., & Lloyd, P. (2001). Eigenvector-like measures of centrality for asymmetric relations. *Social networks*, *23*(3), 191-201.

[16] Borgatti, S. P., & Everett, M. G. (2006). A graph-theoretic perspective on centrality. *Social networks*, *28*(4), 466-484.

[17] Borst, W. N. (1997). Construction of engineering ontologies for knowledge sharing and reuse.

[18] Brandes, U. (2005). *Network analysis: methodological foundations* (Vol. 3418). Springer Science & Business Media.

[19] Brin, S., & Page, L. (1998). The anatomy of a large-scale hypertextual web search engine. *Computer networks and ISDN systems*, *30*(1-7), 107-117.

[20] Burgess, J., & Bruns, A. (2012). (Not) the Twitter election: the dynamics of the# ausvotes conversation in relation to the Australian media ecology. *Journalism Practice*, *6*(3), 384-402.

[21] Calvo-Armengol, A., & Jackson, M. O. (2004). The effects of social networks on employment and inequality. *American economic review*, *94*(3), 426-454.

[22] Chen, H., Chung, W., Xu, J. J., Wang, G., Qin, Y., & Chau, M. (2004). Crime data mining: a general framework and some examples. *computer*, (4), 50-56.

[23] Chen, D., Lü, L., Shang, M. S., Zhang, Y. C., & Zhou, T. (2012). Identifying influential nodes in complex networks. *Physica a: Statistical mechanics and its applications*, *391*(4), 1777-1787.

[24] Chen, D. B., Gao, H., Lü, L., & Zhou, T. (2013). Identifying influential nodes in large-scale directed networks: the role of clustering. *PloS one*, *8*(10), e77455.

[25] Chin, C. H., Chen, S. H., Wu, H. H., Ho, C. W., Ko, M. T., & Lin, C. Y. (2014). cytoHubba: identifying hub objects and sub-networks from complex interactome. *BMC systems biology*, *8*(4), S11.

[26] Cho, H., Gay, G., Davidson, B., & Ingraffea, A. (2007) Social networks, communication styles, and learning performance in a CSCL community, *Computers & Education*, *49*(2), 309–329.

[27] Clauset, A., Arbesman, S., & Larremore, D. B. (2015). Systematic inequality and hierarchy in faculty hiring networks. *Science advances*, *1*(1), e1400005.

[28] Coronicová Hurajová, J., Gago Álvarez, S., & Madaras, T. (2018). On decay centrality in graphs. *Mathematica scandinavica*, *123*(1), 39-50.

[29] Cross, R., & Cummings, J. N. (2004). Tie and network correlates of individual performance in knowledge-intensive work. *Academy of management journal*, *47*(6), 928-937.

[30] Cruz, J. E., Emery, R. E., & Turkheimer, E. (2012). Peer network drinking predicts increased alcohol use from adolescence to early adulthood after controlling for genetic and shared environmental selection. *Developmental psychology*, *48*(5), 1390.

[31] Csardi, G., & Nepusz, T. (2006). The igraph software package for complex network research. *InterJournal, Complex Systems*, *1695*(5), 1-9.

[32] Dangalchev, C. (2006). Residual closeness in networks. *Physica A: Statistical Mechanics and its Applications*, *365*(2), 556-564.

[33] Dawson, S. (2008). A study of the relationship between student social networks and sense of community. *Journal of educational technology & society*, *11*(3), 224-238.

[34] De Laat, M., Lally, V., Lipponen, L., & Simons, R. J. (2007). Investigating patterns of interaction in networked learning and computer-supported collaborative learning: A role for Social Network Analysis. *International Journal of Computer-Supported Collaborative Learning*, *2*(1), 87-103.

[35] de-Marcos, L., García-López, E., García-Cabot, A., Medina-Merodio, J. A., Domínguez, A., Martínez-Herráiz, J. J., & Diez-Folledo, T. (2016). Social network analysis of a gamified e-learning course: Small-world phenomenon and network metrics as predictors of academic performance. *Computers in Human Behavior, 60*, 312-321.

[36] Dhand, A., Harp, J., & Borgatti, S. P. (2014). Leadership in neurology: A social network analysis. *Annals of neurology*, *75*(3), 342-350.

[37] Doncheva, N. T., Assenov, Y., Domingues, F. S., & Albrecht, M. (2012). Topological analysis and interactive visualization of biological networks and protein structures. *Nature protocols*, *7*(4), 670.

[38] Ductor, L., Fafchamps, M., Goyal, S., & van der Leij, M. J. (2014). Social networks and research output. *Review of Economics and Statistics*, *96*(5), 936-948.

[39] Duran-Pinedo, A. E., Paster, B., Teles, R., & Frias-Lopez, J. (2011). Correlation network analysis applied to complex biofilm communities. *PloS one*, *6*(12), e28438.

[40] Ennett, S. T., Bauman, K. E., Hussong, A., Faris, R., Foshee, V. A., Cai, L., & DuRant, R. H. (2006). The peer context of adolescent substance use: Findings from social network analysis. *Journal of research on adolescence*, *16*(2), 159-186.

[41] Estrada, E., Higham, D. J., & Hatano, N. (2009). Communicability betweenness in complex networks. *Physica A: Statistical Mechanics and its Applications*, *388*(5), 764-774.

[42] Everett, M. G., & Borgatti, S. P. (1999). The centrality of groups and classes. *The Journal of mathematical sociology*, *23*(3), 181-201.

[43] Faghani, M. R., & Nguyen, U. T. (2013). A study of XSS worm propagation and detection mechanisms in online social networks. *IEEE Transactions on Information Forensics and Security*, *8*(11), 1815-1826.

[44] Flórez, A. F., Park, D., Bhak, J., Kim, B. C., Kuchinsky, A., Morris, J. H., ... & Muskus, C. (2010). Protein network prediction and topological analysis in Leishmania major as a tool for drug target selection. *BMC bioinformatics*, *11*(1), 484.

[45] Fouss, F., Saerens, M., & Shimbo, M. (2016). *Algorithms and models for network data and link analysis*. Cambridge University Press.

[46] Freeman, L. C. (1977). A set of measures of centrality based on betweenness. *Sociometry*, 35-41.

[47] Freeman, L. C. (1979). Centrality in social networks conceptual clarification. *Social networks*, 1(3), 215-239.

[48] Gašević, D., Zouaq, A., & Janzen, R. (2013). “Choose your classmates, your gpa is at stake!” The association of cross-class social ties and academic performance. *American Behavioral Scientist*, 0002764213479362.

[49] Ghali, N., Panda, M., Hassanien, A. E., Abraham, A., & Snasel, V. (2012). Social networks analysis: Tools, measures and visualization. In *Computational social networks* (pp. 3-23). Springer, London.

[50] Ghazzali, N., & Ouellet, A. (2017, October). Comparative Study of Centrality Measures on Social Networks. In: Dokas, I.M., Bellamine-Ben Saoud, N., Dugdale, J., & Díaz, P. (Eds.) *International Conference on Information Systems for Crisis Response and Management in Mediterranean Countries* (Springer).

[51] Gile, K., & Handcock, M. S. (2006) *Model-based assessment of the impact of missing data on inference for networks* (Seattle, University of Washington).

[52] Gile, K. J., & Handcock, M. S. (2017) Analysis of networks with missing data with application to the National Longitudinal Study of Adolescent Health, *Journal of the Royal Statistical Society: Series C (Applied Statistics)*, *66*(3), 501-519.

[53] Goh, K. I., Kahng, B., & Kim, D. (2001). Universal behavior of load distribution in scale-free networks. *Physical Review Letters*, *87*(27), 278701.

[54] Gräßler, J., Koschützki, D., & Schreiber, F. (2012). CentiLib: comprehensive analysis and exploration of network centralities. *Bioinformatics*, *28*(8), 1178-1179.

[55] Guarino, N., Oberle, D., & Staab, S. (2009). What is an ontology?. In *Handbook on ontologies* (pp. 1-17). Springer, Berlin, Heidelberg.

[56] Guizzardi, G. (2007). On ontology, ontologies, conceptualizations, modeling languages, and (meta) models. *Frontiers in artificial intelligence and applications*, *155*, 18.

[57] Hage, P., & Harary, F. (1995). Eccentricity and centrality in networks. *Social networks*, *17*(1), 57-63.

[58] Hair, J. F., Black, W. C., Babin, B. J., & Anderson, R. E. (2010) *Multivariate Data Analysis: A Global Perspective* (7th ed.) (New Jersey, Pearson Education).

[59] Handcock, M. S., Hunter, D. R., Butts, C. T., Goodreau, S. M., & Morris, M. (2008). statnet: Software tools for the representation, visualization, analysis and simulation of network data. *Journal of statistical software*, *24*(1), 1548.

[60] Hanneman, R. A., & Riddle, M. (2005). *Introduction to social network methods*.

[61] Hommes, J., Rienties, B., de Grave, W., Bos, G., Schuwirth, L., & Scherpbier, a. (2012) Visualising the invisible: A network approach to reveal the informal social side of student learning, *Advances in Health Sciences Education*, 17(5), 743–757.

[62] Hubbell, C. H. (1965). An input-output approach to clique identification. *Sociometry*, 377-399.

[63] Huisman, M. (2009) Imputation of missing network data: some simple procedures, *Journal of Social Structure*, *10*(1), 1-29.

[64] Huisman, M., Krause, R. W., Alhajj, R., & Rokne, J. (2018). Imputation of Missing Network Data.

[65] Hunter, D. R., Handcock, M. S., Butts, C. T., Goodreau, S. M., & Morris, M. (2008). ergm: A package to fit, simulate and diagnose exponential-family models for networks. *Journal of statistical software*, *24*(3).

[66] Iacobucci, D., Henderson, G., Marcati, A., & Chang, J. (1996). Network analyses of brand switching behavior. *International Journal of Research in Marketing*, *13*(5), 415-429.

[67] Idowu, O. C., Lynden, S. J., Young, M. P., & Andras, P. (2004, August). *Bacillus Subtilis protein interaction network analysis*. In Proceedings of the 2004 IEEE Computational Systems Bioinformatics Conference (CSB 2004) (pp. 623-625).

[68] Joy, M. P., Brock, A., Ingber, D. E., & Huang, S. (2005). High-betweenness proteins in the yeast protein interaction network. *BioMed Research International*, *2005*(2), 96-103.

[69] Joyce, K. E., Laurienti, P. J., Burdette, J. H., & Hayasaka, S. (2010). A new measure of centrality for brain networks. *PloS one*, *5*(8), e12200.

[70] Jarumaneeroj, P. (2014). *An economics study of container ports in the global network of container shipping* (Doctoral dissertation, Georgia Institute of Technology).

[71] Katona, Z., Zubcsek, P. P., & Sarvary, M. (2011). Network effects and personal influences: The diffusion of an online social network. *Journal of marketing research*, *48*(3), 425-443.

[72] Katz, L. (1953). A new status index derived from sociometric analysis. *Psychometrika*, *18*(1), 39-43.

[73] Kelly, S. T. (2017). *A Bioinformatics Approach to Synthetic Lethal Interactions in Cancer with Gene Expression Data* (Doctoral dissertation). University of Otago.

[74] Kelly, S. T. (2019). *TomKellyGenetics/info.centrality: an R implementation of information centrality using igraph*. Retrieved from https://rdrr.io/github/TomKellyGenetics/info.centrality/

[75] Kim, H. K., Kim, J. K., & Chen, Q. Y. (2012). A product network analysis for extending the market basket analysis. *Expert Systems with Applications*, *39*(8), 7403-7410.

[76] Kiss, C., & Bichler, M. (2008). Identification of influencers—measuring influence in customer networks. *Decision Support Systems*, *46*(1), 233-253.

[77] Kleinberg, J. M. (1999a). Authoritative sources in a hyperlinked environment. *Journal of the ACM (JACM)*, *46*(5), 604-632.

[78] Kleinberg, J. M. (1999b). Hubs, authorities, and communities. *ACM computing surveys (CSUR)*, *31*(4es), 5.

[79] Kolaczyk, E.D., & Gábor, C. (2014) *Statistical analysis of network data with R*. (Vol. 65.) (New York: Springer).

[80] Korn, A., Schubert, A., & Telcs, A. (2009). Lobby index in networks. *Physica A: Statistical Mechanics and its Applications*, *388*(11), 2221-2226.

[81] Koschützki, D., & Schreiber, F. (2008). Centrality analysis methods for biological networks and their application to gene regulatory networks. *Gene regulation and systems biology*, *2*, GRSB-S702.

[82] Kossinets, G. (2006) Effects of missing data in social networks, *Social networks*, *28*(3), 247-268.

[83] Krackhardt, D. (1990). Assessing the political landscape: Structure, cognition, and power in organizations. *Administrative science quarterly*, 342-369.

[84] Krebs, V. (2002). Uncloaking terrorist networks. *First Monday*, *7*(4).

[85] Landherr, A., Friedl, B., & Heidemann, J. (2010). A critical review of centrality measures in social networks. *Business & Information Systems Engineering*, *2*(6), 371-385.

[86] Lee, S. H., Cotte, J., & Noseworthy, T. J. (2010). The role of network centrality in the flow of consumer influence. *Journal of Consumer Psychology*, *20*(1), 66-77.

[87] Lewis, J. M. (2006). Being around and knowing the players: networks of influence in health policy. *Social Science & Medicine*, *62*(9), 2125-2136.

[88] Lin, N. (1976). *Foundations of social research*. McGraw-Hill Companies.

[89] Lin, C. Y., Chin, C. H., Wu, H. H., Chen, S. H., Ho, C. W., & Ko, M. T. (2008). Hubba: hub objects analyzer—a framework of interactome hubs identification for network biology. *Nucleic Acids Research*, 1-6.

[90] Liu, Z., Kang, L., Domanska, M., Liu, S., Sun, J. and Fang, C (2018). *Social network characteristics of learners in a course forum and their relationship to learning outcomes*. In Proceedings of the 10th International Conference on Computer Supported Education (CSEDU 2018) - Volume 1, pages 15-21.

[91] Lü, L., Chen, D., Ren, X. L., Zhang, Q. M., Zhang, Y. C., & Zhou, T. (2016). Vital nodes identification in complex networks. *Physics Reports*, *650*, 1-63.

[92] Malhotra, N., Décaudin, J. M., & Bouguerra, A. (2007) *Etudes Marketing avec SPSS* (5ème édition) (France, Pearson Education).

[93] Mehra, A., Kilduff, M., & Brass, D. J. (2001). The social networks of high and low self-monitors: Implications for workplace performance. *Administrative science quarterly*, *46*(1), 121-146.

[94] Mersch, D. P. (2016). The social mirror for division of labor: what network topology and dynamics can teach us about organization of work in insect societies. *Behavioral ecology and sociobiology*, *70*(7), 1087-1099.

[95] Montgomery, J. D. (1991). Social networks and labor-market outcomes: Toward an economic analysis. *The American economic review*, *81*(5), 1408-1418.

[96] Mushtaq, A., Badar, K., Anwar, M., & Abbas, S. G. (2016). Exploring the relationship of network centrality and academic performance of female students. *Sarhad Journal of Management Sciences*, *2*(2), 195–206.

[97] Neal, Z. P. (2008). The duality of world cities and firms: comparing networks, hierarchies, and inequalities in the global economy. *Global Networks*, *8*(1), 94-115.

[98] Obadi, G., Drázdilová, P., Martinovic, J., Slaninová, K., & Snásel, V. (2010). Using spectral clustering for finding students' patterns of behavior in social networks. In *DATESO* (pp. 118–130).

[99] Ortiz-Arroyo, D., & Hussain, D. A. (2008, December). An information theory approach to identify sets of key players. In *European Conference on Intelligence and Security Informatics* (pp. 15-26). Springer, Berlin, Heidelberg.

[100] Mastrobuoni, G., & Patacchini, E. (2012). Organized crime networks: An application of network analysis techniques to the American mafia. *Review of Network Economics, 11*(3).

[101] Page, L., Brin, S., Motwani, R., & Winograd, T. (1999). *The PageRank citation ranking: Bringing order to the web*. Stanford InfoLab.

[102] Pal, S. K., Kundu, S., & Murthy, C. A. (2014). Centrality measures, upper bound, and influence maximization in large scale directed social networks. *Fundamenta Informaticae*, *130*(3), 317-342.

[103] Peng, X., Wang, J., Wang, J., Wu, F. X., & Pan, Y. (2015). Rechecking the centrality-lethality rule in the scope of protein subcellular localization interaction networks. *PloS one*, *10*(6), e0130743.

[104] Perry-Smith, J. E. (2006). Social yet creative: The role of social relationships in facilitating individual creativity. *Academy of Management journal*, *49*(1), 85-101.

[105] Pfeffer, J., & Carley, K. M. (2012, April). *k-centralities: Local approximations of global measures based on shortest paths*. In Proceedings of the 21st International Conference on World Wide Web (pp. 1043-1050). ACM.

[106] Poldin, O., Valeeva, D., & Yudkevich, M. (2016). Which peers matter: How social ties affect peer-group effects. *Research in Higher Education*, *57*(4), 448-468.

[107] Pržulj, N., Wigle, D. A., & Jurisica, I. (2004). Functional topology in a network of protein interactions. *Bioinformatics*, *20*(3), 340-348.

[108] Qi, X., Fuller, E., Wu, Q., Wu, Y., & Zhang, C. Q. (2012). Laplacian centrality: A new centrality measure for weighted networks. *Information Sciences*, *194*, 240-253.

[109] Rdocumentation. (2017). *centiserve v1.0.0*. Retrieved September 17, 2019 from https://www.rdocumentation.org/packages/centiserve/versions/1.0.0

[110] rdrr.io. (2019). *centiserve: Find Graph Centrality Indices*. Retrieved September 17, 2019 from https://rdrr.io/cran/centiserve/

[111] Robins, G., Pattison, P., & Woolcock, J. (2004) Missing data in networks: Exponential random graph (p∗) models for networks with non-respondents, *Social networks*, *26*(3), 257-283.

[112] Russo, T. C., & Koesten, J. (2005). Prestige, centrality, and learning: A social network analysis of an online class. *Communication Education*, *54*(3), 254-261.

[113] Saqr, M., Fors, U., & Tedre, M. (2018a). How the study of online collaborative learning can guide teachers and predict students' performance in a medical course. *BMC medical education, 18*(1), 24.

[114] Saqr, M., Fors, U., & Nouri, J. (2018b). Using social network analysis to understand online Problem-Based Learning and predict performance. *PloS one*, *13*(9), e0203590.

[115] Seidman, S. B. (1983). Network structure and minimum degree. *Social networks*, *5*(3), 269-287.

[116] Shen, D., Nuankhieo, P., Huang, X., Amelung, C., & Laffey, J. (2008). Using social network analysis to understand sense of community in an online learning environment. *Journal of Educational Computing Research*, *39*(1), 17-36.

[117] Shimbel, A. (1953). Structural parameters of communication networks. *The bulletin of mathematical biophysics*, *15*(4), 501-507.

[118] Song, Z., Sun, Y., Yi, J., & Ni, L. (2015). Methods of importance evaluation for information subsystems in manufacturing enterprises based on centrality. *Open Journal of Business and Management*, *3*(02), 125.

[119] Sparrowe, R. T., Liden, R. C., Wayne, S. J., & Kraimer, M. L. (2001). Social networks and the performance of individuals and groups. *Academy of management journal*, *44*(2), 316-325.

[120] Thomas, S. L. (2000) Ties that bind: A social network approach to understanding student integration and persistence, *Journal of Higher Education*, *71*(5), 591–615.

[121] Tsai, W. (2001). Knowledge transfer in intraorganizational networks: Effects of network position and absorptive capacity on business unit innovation and performance. *Academy of management journal*, *44*(5), 996-1004.

[122] Tufféry, S. (2012) *Data Mining et statistique décisionnelle: L'intelligence des données* (Paris, Éditions Technip).

[123] Valente, T. W., Coronges, K., Lakon, C., & Costenbader, E. (2008). How correlated are network centrality measures?. *Connections*, *28*(1), 16.

[124] Vargas, D. L., Bridgeman, A. M., Schmidt, D. R., Kohl, P. B., Wilcox, B. R., & Carr, L. D. (2018). Correlation between student collaboration network centrality and academic performance. *Physical Review Physics Education Research*, *14*(2), 020112.

[125] Vaughan, S., Sanders, T., Crossley, N., O'neill, P., & Wass, V. (2015) Bridging the gap: The roles of social capital and ethnicity in medical student achievement, Medical Education, 49(1), 114–123.

[126] Viswanath, M. (2009). *Ontology-based automatic text summarization* (Doctoral dissertation). uga.

[127] Wang, J., Li, M., Wang, H., & Pan, Y. (2011). Identification of essential proteins based on edge clustering coefficient. *IEEE/ACM Transactions on Computational Biology and Bioinformatics*, *9*(4), 1070-1080.

[128] Wang, C., Butts, C. T., Hipp, J. R., Jose, R., & Lakon, C. M. (2016) Multiple imputation for missing edge data: A predictive evaluation method with application to Add Health. *Social networks*, *45*, 89-98.

[129] Wasserman, S., & Faust, K. (1994) *Social Network Analysis: Methods and Applications* (8th ed.) (Cambridge, Cambridge University press).

[130] White, S., & Smyth, P. (2003, August). Algorithms for estimating relative importance in networks. In *Proceedings of the ninth ACM SIGKDD international conference on Knowledge discovery and data mining* (pp. 266-275). ACM.

[131] Wiener, H. (1947). Structural determination of paraffin boiling points. *Journal of the American Chemical Society*, *69*(1), 17-20.

[132] Wolfram Research. (2015). *Radiality Centrality*. Retrieved from http://reference.wolfram.com/language/ref/RadialityCentrality.html

[133] Woolf, K., Potts, H. W., Patel, S., & McManus, I. C. (2012). The hidden medical school: a longitudinal study of how social networks form, and how they relate to academic performance. Medical teacher, 34(7), 577-586.

[134] Xu, W. W., Sang, Y., Blasiola, S., & Park, H. W. (2014). Predicting opinion leaders in Twitter activism networks: The case of the Wisconsin recall election. *American Behavioral Scientist*, *58*(10), 1278-1293.

[135] Xu Li, X., Yin, J., Tang, J., Li, Y., Yang, Q., Xiao, Z., ... & Xue, W. (2018). Determining the balance between drug efficacy and safety by the network and biological system profile of its therapeutic target. *Frontiers in pharmacology*, *9*.

[136] Yang, H., & Tang, J. (2003) Effects of social network on students' performance: a web-based forum study in Taiwan, *Journal of Asynchronous Learning Networks*, *7*(3), 93–107.

[137] Yang, D., Sinha, T., Adamson, D., & Rosé, C. P. (2013, December). *Turn on, tune in, drop out: Anticipating student dropouts in massive open online courses*. In Proceedings of the 2013 NIPS Data-driven education workshop (Vol. 11, p. 14).

[138] Yu, H., Kim, P. M., Sprecher, E., Trifonov, V., & Gerstein, M. (2007). The importance of bottlenecks in protein networks: correlation with gene essentiality and expression dynamics. *PLoS computational biology*, *3*(4), e59.

[139] Zedan, S., & Miller, W. (2017). Using social network analysis to identify stakeholders' influence on energy efficiency of housing. *International Journal of Engineering Business Management*, *9*, 1847979017712629.

[140] Zhang, X., Venkatesh, V., & Huang, B. (2008) *Students interactions and course performance: Impacts of online and offline networks*. Proceedings of the 2008 International Conference on Information System (Paper 215).

[141] Zhang, R., & Lin, Y. (2008). DEG 5.0, a database of essential genes in both prokaryotes and eukaryotes. *Nucleic acids research*, *37*(suppl_1), D455-D458.

[142] Zhang, Y., Wang, Z., & Xia, C. (2010, April). *Identifying key users for targeted marketing by mining online social network*. In 2010 IEEE 24th International Conference on Advanced Information Networking and Applications Workshops (pp. 644-649). IEEE.

[143] Zhang, K., Zhang, H., dong Wu, Y., & Bao, F. (2011, December). *Evaluating the importance of nodes in complex networks based on principal component analysis and grey relational analysis*. In Networks (ICON), 17th IEEE International Conference (pp. 231-235).

[144] Zhang, X., & Venkatesh, V. (2013). Explaining employee job performance: The role of online and offline workplace communication networks. Mis Quarterly, 695-722.

[145] Žnidaršič, A., Ferligoj, A., & Doreian, P. (2012) Non-response in social networks: The impact of different non-response treatments on the stability of blockmodels, *Social Networks*, *34*(4), 438-450.

[146] Zwolak, J. P., Dou, R., Williams, E. A., & Brewe, E. (2017) Students' network integration as a predictor of persistence in introductory physics courses, *Physical Review Physics Education Research*, *13*(1), 10113.

 Click here to download Figure Fig 1.png

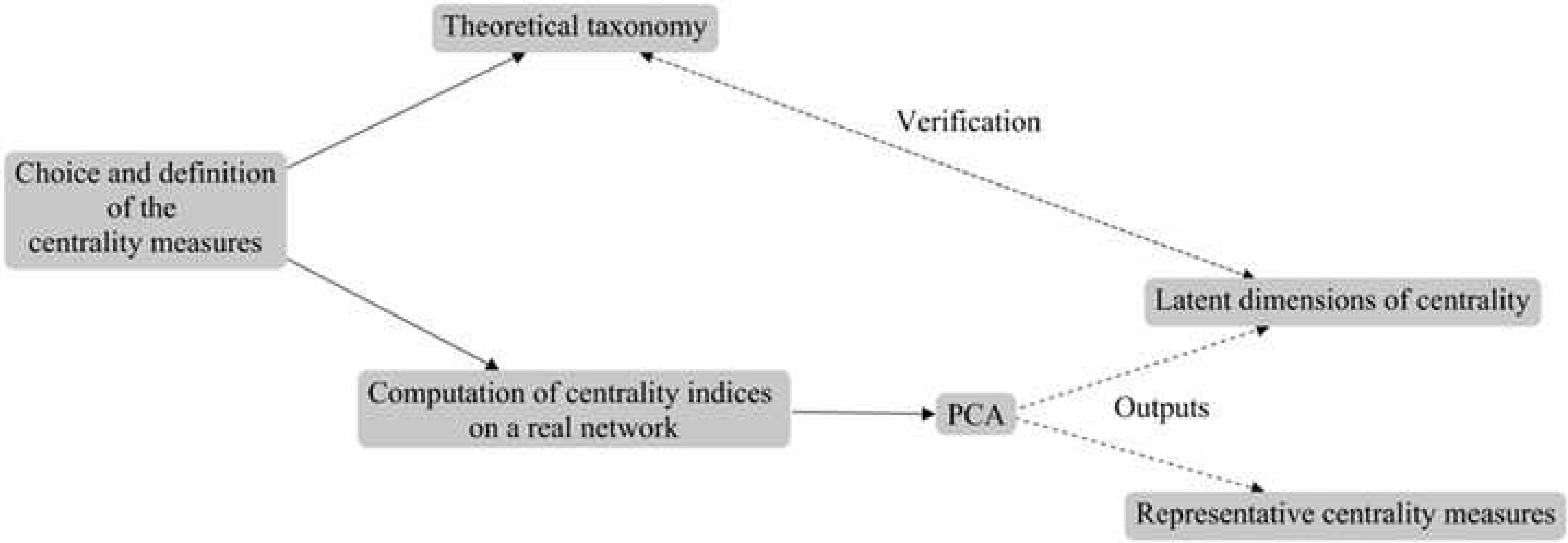

Figure 2 Click here to download Figure Fig 2.png

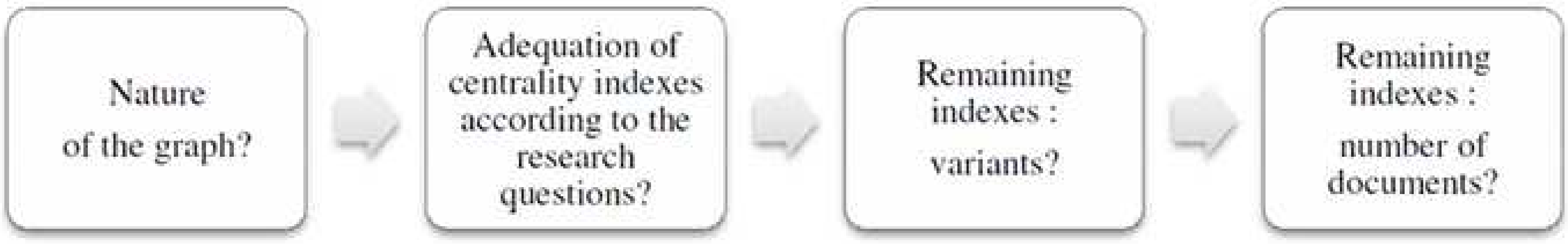

Appendix 1

Click here to access/download
Supporting Information
Taxonomy_Appendix1_Indices List.docx

Click here to access/download
Supporting Information
Taxonomy_Appendix2_definitions.docx
