# Supplementary material for: A methodology and theoretical taxonomy for centrality measures: What are the best centrality indicators for student networks?
Source: PLoS One. 2020 Dec 30;15(12):e0244377. doi: 10.1371/journal.pone.0244377 (PMC7773201; doi:10.1371/journal.pone.0244377)
Supplement: Attachment — Submitted filename: Response to Reviewers.docx [file pone.0244377.s010.docx]

Response to Reviewers

We first would like to thank the two reviewers and the editor most warmly for the very constructive comments that were made and enabled us to improve the paper.

For more easiness when proofreading the paper, the comments and action that were taken are organized according to the different ordered parts of the manuscript in Table 1. Also, the modifications, the additions, etc., are highlighted in yellow in the revised manuscript.

The additional actions that have been taken according to the recommendations in: ‘*PONE-D-20-06951_reviewer.pdf*’ (from Reviewer 2) - and having not been previously treated in Table 1 - are explained in Table 2 and are also highlighted in yellow in the revised manuscript. A majority of recommendations in ‘*PONE-D-20-06951_reviewer.pdf*’ were already been treated in Table 1.

**Table 1. Comments and action taken according to the different ordered parts of the manuscript.**

| N° | Comment(s) | From | Action(s) |
| --- | --- | --- | --- |
| 1 | There have been concerns that “the present submission is closely related to previously published and freely-accessible work including: *XXX*^[1]^. I would like you to comment on the issue, specifically addressing that “that the separation into more than one article has not compromised the robustness of the statistical analysis (e.g. by removing required adjustments for multiple hypothesis testing).”  *We noted in your submission details that a portion of your manuscript may have been presented or published elsewhere. Please clarify whether this publication was peer-reviewed and formally published. In the cover letter please provide the reason that this work does not constitute dual publication and should be included in the current manuscript.* | Editor | The reasons that this work does not constitute dual publication are explained in the cover letter. |
| 2 | **Abstract:** The problem statement in the first two sentences of the abstract should be made more explicit. It is not clear that the way current SNA methods are applied is actually problematic. What does it mean for the field? And why is it relevant? (this information is mentioned later in the manuscript, but should be included in the abstract). | Reviewer 1 | The abstract was reworked in order to explain the problem statement and the relevance of our research better (see the passages highlighted in yellow in the revised manuscript)   - Rows 26-31 - Rows 43-44 |
| 3 | **Section I. Introduction.**  While there is a rather clear problem statement presented in the introduction, the research rationale behind this study is missing. This needs to be included in the introduction. Consider rewriting this part. The literature review that follows must be separate from the introduction, and not be a sub-part of it. While the two research aims are presented in the different sections of the introduction (1. “develop a methodology that verifies the proposed theoretical taxonomy on real data”, 2. “selecting the best suited centrality measures for representing a student network or for studying the impacts of student networks on education outcomes such as student performance”), they are missing motivation and rationale. When re-writing this section, specify 1) problem statement, 2) motivation (what, how and why), 3) approach In section 1.5. the approach on how to tackle the problem and achieving the goals is missing. This section generates more questions than it answers. | Reviewer 1 | 1) ‘*Consider rewriting the introduction*’: the introduction was rewritten and follows the following sequence: problem statement (rows 54-60); motivation: what (rows 60-66); motivation: why (rows 68-112, with the explicit motivations highlighted in yellow in the text); approach: how to tackle the problem and achieving the goals (rows 114-124); organization of the paper (rows 126-133). 2) *‘The literature review that follows must be separate from the introduction, and not be a sub-part of it’*: a theoretical section *(II. Theoretical background: Centrality measures and taxonomies for student networks*) was added to separate the literature review from the introduction. |
| 4 | **Section I. Introduction (1.1):**The relevance of centrality measures in networks  The first part of this section is relevant. The second part is a list of applications, but without a clear purpose. Make sure that this list at least adds something to the research that is presented, i.e. make the links between the literature and your study more explicit. | Reviewer 1 | The section is now included in :  ***II. Theoretical background: Centrality measures and taxonomies for student networks***,  Section 2.1. *‘The relevance and use of centrality measures within (student) networks’*.  The primary purpose of the list of applications was to highlight the importance of centrality within network studies, whatever the types of graph considered. It was however simplified, since our research indeed applies our proposed methodology only to a specific friendship student network. Highlighting centrality within networks by means of those various applications introduces the centrality question within specific student networks. Then, in order to make the links between the literature and our study more explicit, we added an Appendix (Appendix 1) that cites and computes the number and percentages of the centrality measures that are used for all the referenced studies (for student networks and other networks), to support the statement that mostly the same indices are used, whatever the type of network considered (rows 167-176) |
| 5 | **Section I. Introduction (1.2. & 1.3):**  In this section the research rationale is presented. Elaborate on the relevance of your work. Go beyond the fact that there hasn’t been done any research on this topic; rather explain why there should have been.  The relevance of the use of ontology and taxonomy should be substantiated much more. Why are these relevant, and in particular, why is the theoretical taxonomy relevant? The reason I ask is because I do not see how the theoretical taxonomy explains network centrality measure in terms of e.g. student learning. So, if it is important that this is a *student* network, then the authors should spend more effort on connecting their student network to issues relating to student learning or student education in general. | Reviewer 1  Reviewer 2 | The section is now included in:  ***II. Theoretical background: Centrality measures and taxonomies for student networks***,  Section 2.2. *‘A theoretical taxonomy of centrality measures for student networks’*.  The elaborated rationale, together with the usefulness of a theoretical classification of centrality measures, especially for student networks, is highlighted in yellow in the text (rows 232-251). |
| 6 | **Section III. The proposal of an integrated methodology for studying centrality within networks**  Obviously, when the authors choose particular network centrality measures, as a reader, I will look for reasons why. I have not found convincing reasons for the first “proper centralities” nor for the 18 chosen centralities. Particularly since the authors claim that they have made a theoretical consideration based on the nature of the network, and I was not able to find these considerations in the text. | Reviewer 2 | See section 3.1. ‘*Choosing and defining the centrality measures*’: we explained the choice - related to the fact that we work on a friendship student network - of the centrality measures, for each of the eighteen indices (rows 322 - 422) |
| 7 | **Section 3.4: The PCA as methodological tool:**  Another methodological choice is that of PCA. Was this the choice made by the few others who have tried to cluster centrality measures? Why not perform a PCA on all the “proper” centrality measures? Did that violate the KMO-criterion of 0.5?  **+ Comments in the PONE-D-20-06951_reviewer.pdf :**  ‘*Fourth, a PCA is applied on the computed centrality measures*’  Comments: What is your methodological reasoning behind using PCA? How will that connect your data to your theoretical proposition?  ‘*PCA enables for identifying the factor on which a variable* ***saturates*** *the most*’  Comment: Interesting word - do you mean where the variable has the highest loading?  ‘*We performed PCA to determine the most representative centrality measures*’  Comment: How did you use PCA to identify the most representative measures? | Reviewer 2 | 1) *Another methodological choice is that of PCA. Was this the choice made by the few others who have tried to cluster centrality measures?*  - We added references of studies that also used PCA and/or other techniques (see rows 451-459) - We justified why, in our case, PCA is the most relevant choice, i.e., yields answers to our research questions (see rows 476-486).  2) *Why not perform a PCA on all the “proper” centrality measures? Did that violate the KMO-criterion of 0.5?*  - We performed PCA on all eighteen centrality measures that we chose for this paper (in the text: ‘*we performed PCA on our eighteen centrality indices*’) by using the correlation matrix in order to deal with the different measurement scales for the centrality measures (in the text: ‘*worked on standardized data to neutralize the problem of centrality measures with different units*’) (see rows 469-471).  3) *‘On which a variable* ***saturates*** *the most’: Do you mean where the variable has the highest loading?*  - Yes. We replaced the word ‘*saturates*’ by your proposal in the revised text (see row 479 + row 585 in the *results* section).  4) *How did you use PCA to identify the most representative measures?*  - In the original manuscript, this was explained in Section 4.3. We moved the theoretical explanation to Section 3.4. (rows 486-500), and provide a brief reminder of the method in Section 5.4. (see rows 691-695) |
| 8 | p. 22 “*We applied the integrated methodology that we developed - i.e., (1) the choice, definition and proposal of a theoretical classification of centrality measures, (2) the highlighting of centrality dimensions within a network of interest, (3) the verification of the proposed theoretical taxonomy by means of those dimensions, and (4) the identification of representative centrality indices - on a real student network.*” Yet, the steps you undertook are not elaborated upon extensively in the paper. Link this to your own data set. Detail the choices you made when analyzing the student network in the methodology and results sections. | Reviewer 1 | 1) **The steps are elaborated in:**   **Section III (Methodology section)**  The section’s introduction has been reworked in order to explain the integrated methodology better (rows 255-265). Then, the steps of the methodology, together with the links to our data set, are elaborated in the following sub-sections:  3.1. ‘*Choosing and defining the centrality measures*’: The section has been reworked in order to justify the choice of centrality indices better, in our case for our own friendship student network: see in comment number 6 of reviewer 2, and the passages highlighted in yellow in the section. Figure 3 was also slightly adapted to illustrate better the sequence followed within the methodology first step.  3.2. ‘*Proposal of a theoretical taxonomy*’. The theoretical proposal is generic and not linked to any network in particular, but related to criteria of centrality measures (we however added rows 426 & 429-433 in the section to be more specific).  3.3. ‘*Computation of the centrality measures on a real network*’  3.4. ‘*The Principal Component Analysis as a methodological tool*’: The section has been reworked in order to explain the choice of the PCA as methodological tool better; see in comment number 7 of reviewer 2.  **2) The analyses on our student network are detailed in:**  **Section V (Result section)**  5.1. ‘*Correlations between the centrality measures*’  5.2. ‘*The centrality latent dimensions within friendship student networks*’: This section highlights the latent dimensions of centrality that were found within our friendship student network, by means of the PCA results.  5.3. ‘*The verification of the taxonomy on real data: a friendship student network*’: This section verifies the theoretical and generic taxonomy on real data: our friendship student network, also by means of the PCA results.  5.4. ‘*The most representative measures of centrality for friendship student networks*’: this section highlights the most representative and significant indices when investigating and representing friendship student networks. |
| 9 | **Section IV: Data:** I find the imputation step you have done to account for the missing values not fully explained, I am missing the ERGM formula, the terms used, the diagnostics and GOF. It might be good if you include a summary of these data in the text, and the full details in an appendix. | Editor | See Appendix 5 for the details, and the summary in the text (see rows 522-532) |
| 10 | **Section IV: Data:** Explain in more detail what type of data you used: mode (written, online, …) and way of collecting the data. Also mention the number of nodes and ties in the network. This is interesting for researchers who want to apply your findings to their own study. | Reviewer 1 | The type of data: their mode, the way of collecting them, and the number of nodes and ties in the network are added in the passages highlighted in yellow in the revised manuscript (rows 504-509, rows 515-518 and rows 527-528). |
| 11 | **Section V: Results:** Table 2. Specify: coefficients for which the p-value is ≤ 0. (seems to be incomplete) | Reviewer 1 | Above the table: ‘*In bold font: coefficients for which the p-value is ≤ 0*’ |
| 12 | **Section VI: Discussion:** Mind repetition in the limitations | Reviewer 1 | We deleted the repetitions within the paragraph that concerned the limitations. |
| 13 | On p. 19 “*Yet based on shortest paths in their algorithm, bottlenecks seem therefore to measure a different type of centrality than the residual closeness, geodesic k-path and betweenness indices.*” This requires an explanation. (This is repeated on p. 24 …) | Reviewer 1 | **Section VI. Discussion**: We proposed an hypothesis that could explain why the bottleneck centrality seems to cover a particular type of centrality (rows 774-761). |
| 14 | One thing that I would find important to theoretical considerations is the name generator and what a connection might mean. Since it is a friendship, any flow of information is inferred. Friendships can mean many things, and some friendships might not consist of sharing of information relevant to e.g. learning on education — even if the friendship is between two students. From the work I have participated, the nature of connections was important to which centrality measures best predicted later grades. | Reviewer 2 | 1) The scope of the article was reduced to friendship student networks, instead of ‘*general*’ student networks (student networks replaced by friendship student networks in the revised manuscript). 2) In Section 2.1. (*The relevance and use of centrality measures within (student) networks*), we emphasized that the nature of the investigated ties might indeed also affect the impact of centrality on educational outcomes (rows 183-185). 3) Section ‘**Discussion’**: We added that our study should be replicated for other types of ties investigated within student networks, since on the one hand centrality within different types of links has diverse advantages for educational outcomes, and on the other hand, the representativeness of centrality might be different according to the nature of the ties that are considered (rows 901-904 and 910-924). |
| 15 | Section “2.1. Choosing and defining the centrality measures”  You have used the centrality measured offered by one R library, there are of course others offered by other libraries, in such an article where you are offering a taxonomy, you may need to be comprehensive, include - in your background or/in your analysis- other centrality measures that are commonly used and not offered by CINNA (Centiserver, keyplayer, igraph), | Editor | Jalili & al. (2015) (reference added) identified 113 centrality measures (rows 927-929). It is impossible for us to include other measures, due to lack of time and resources and in order to keep the paper to a reasonable length.   - The scope of the paper was reduced to one library (CINNA) and eighteen indices throughout the entire manuscript (e.g., see abstract, rows 37-38). - In Section 3.1. ‘*Choosing and defining the centrality measures*’: We justified the choice of the eighteen centrality measures (see comment number 6 of reviewer 2) - Section ‘**Discussion’**: We emphasized the fact that future studies could replicate our methodology on other indices and other libraries (rows 896-899 and rows 931 - 933) |
| 16 | Generalizations of findings. I believe that it is very important that the authors are investigating a single network. This means that the taxonomy is not general but needs verification using other networks. It is mentioned in the end, but it should be clear from the beginning that this is a particular network with a particular name generator, particular students in a particular setting. The authors could expand a little on the generalizability by applying their methodology on the network without the ~300 non-responding students as well as the impute-connections network. If they find roughly the same rotated components, then this may be a good sign. | Reviewer 2 | 1) As stated in regard to comment 14, the scope of the paper was reduced to one single friendship student network throughout the entire manuscript (e.g., see abstract, row 37). 2) Section ‘**Discussion’**:  - We emphasized the need to verify the taxonomy on other student networks, but also on other types of graph (rows 993-996) - In order to generalize our findings, as recommended, we computed the eighteen centrality indices on the *original* network (i.e., on the 574 respondents only), and applied the PCA to those centrality measures computed for those 574 students. Then, we compared the components that came from the PCA carried out on the 574 respondents only with the latent dimensions highlighted from the PCA performed on the augmented network (i.e., the 870 students): rows 852–890 and Appendix 6. |
| 17 | Rework the conclusion. This section is a summary of the aims, but not of the work that has been done. **Summarize the findings**, rather than stating that you fulfilled the aims of the research study.  The same goes for the final sentence: state your main findings and **possible recommendations**. | Reviewer 1 | - The main findings are summarized in rows 944-960. - The possible recommendations are stated in rows 960-963. |
| 18 | Please let the manuscript be proofread before resubmission. | Reviewer 1 | The revised manuscript has been proofread by a professional scientific translator. |
| 19 | Please upload the minimal anonymized data set necessary to replicate your study findings as Supporting Information files. | Journal requirement | The 2 data files (i.e., for the original network and for the augmented network), which each contain the eighteen computed centrality indices, will be held - after acceptance - in a public repository, include URLs, accession numbers or DOIs.  Please contact Michelle Remy (michele.remy@uclouvain.be) if there is a need to complete an official document. |
| 20 | Please proofread for numerical typos (commas instead of decimal points) | Journal requirement | The commas were replaced by decimal points within the entire revised manuscript and within the concerned appendices. |
| 21 | If applicable you deposit your laboratory *protocols* in protocols.io | Journal requirement | Our study is not laboratory research. |
| 22 | Thank you for including your ethics statement on the online submission form: "The study concerns human subjects  The juridic department of the USL-B approved the study  The survey contained all the required information for an informed consent and the survey was not mandatory  the data were anonymized before the analyses". To help ensure that the wording of your manuscript is suitable for publication, would you please also add this statement at the beginning of the Methods section of your manuscript file. | Journal requirement | See footnote page 19 of the revised manuscript. |

**Table 2. Additional changes or remarks based on the recommendations in: ‘*PONE-D-20-06951_reviewer.pdf*’**

| N° of the comment (PONE-D-20-06951) | N° of the page (PONE-D-20-06951) | Comment(s) | Action(s) and rows in the revised manuscript |
| --- | --- | --- | --- |
| 1-5 | 3 | Introduction section :   - I think you should start with some of the text in 1.1, but before that have a couple of sentences about what the topic will be and why it is important on a broad scale. - This paragraph appears largely as unsubstantiated claims. I would either remove it or - Why this crucial, why is is it first? - Yes, but why would you do that? Are there studies which would indicate that such a strategy is - highlighted text | The introduction has been entirely rewritten (pages 3-5 in the revised manuscript), and the rationales behind the study were added (see comment number 3 and actions taken in Table 1) |
| 6 | 3 | ‘*In SNA, a major issue concerns how the nodes are characterized within a network*’ 🡺 Says who? | - Reformulated: rows 142-143 - Reference added. |
| 7 | 3 | ‘*A network is a set of connected nodes*’ 🡺 could be more precise. Usually, a set of nodes and a set of links connecting these nodes. | - Reformulated: row 140 |
| 8 | 3 | ‘*The centrality concept is one of the*  *most powerful indicators*’ 🡺 More powerful than what, and says who? | - Reformulated: rows 142-143 - Reference added. |
| 9 | 4 | From your title, as a reader, I am expecting to hear about student networks, and you have no references to that part of the literature here. I think you should have that here as well. | The section was reworked and now elaborated in Section 2.1.(see comment number 4 and actions taken in table 1) |
| 10 | 4 | ‘*One of the objectives of this paper is to thoroughly define a number of centrality*’ 🡺 why is this important? Costa et al, Wasserman & Faust and many others define these measures in detail. Are their definitions not thorough? If so, why not? | The importance of defining and explaining the centrality indices in the process of the methodology that we proposed is described in several parts of the revised manuscript:   - Rows 72-75 - Row 118 - Rows 257-259 - **Rows 286-296** - Row 426 - Rows 432-433 - Row 587 |
| 11 & 12 | 4 | ‘*Another purpose concerns the comparison and the theoretical classification of centrality indices according to several criteria such as their formula*’ 🡺 of what? where do these criteria come from? Why is the classification necessary? | 1) Necessity of the classification :  - Rows 77-81 - Rows 90-91 - Rows 232-251 (see comment 5 and actions taken in table 1)  2) Used criteria for classifying the centrality indices:  - Rows 82-85 - Rows 429-432 |
| 13 | 5 | ‘*such taxonomies of centrality measures are rare in the literature*’ 🡺 Put in a reference here. | It is a statement that we made because we did not find a lot of classifications of centrality measures in the literature. We therefore replaced the initial sentence by ‘*As far as we know, such theoretical taxonomies of centrality measures are rare in the literature*’ (rows 80-81). |
| 14 | 5 | ‘*theoretical classification’* 🡺 What do you mean with "theoretical"? | ‘*i.e.,not yet empirically verified*’: added in rows 78-79 |
| 15 & 16 | 5 | Section 2.2., rows 199-203 (revised manuscript): What do you mean by ontology?  Both taxonomy and ontology are a much broader concepts than in the way they are used here. For example, ontology is used in philosophy of science, and taxonomy is used in educational science (Bloom's taxonomy and SOLO taxonomy). So, if it is important to use it here, you need to tell the reader exactly why a taxonomy is an ontology. Also, it is unclear what is meant by "knowledge engineering". | See Section 2.2: rows 199-203   - In the revised manuscript, we no longer talk about ontology, but only about taxonomies, since we work on classifying objects only. - We state that taxonomies are used in a variety of fields. - We provide a clearer explanation of the concept of taxonomies, including their knowledge contribution. - We deleted the concept of *knowledge engineering*, since the engineering field is included is the application list for taxonomies. |
| 17 | 5 | Section 2.2., rows 206-230 (revised manuscript): this section should be better referenced to make clear from where these postulates originate. Is it all from Guizzardi (2007)? What is the Having read e.g. Guizzardi's (2007) notion of 'soundness' I do not agree with this wording. Guizzardi speaks of modeling primitives and *not* underlying reality. Also, Guizzardi is careful to say that ontology has different meanings in computer sciences and in philosophy .This is not reflected here, even if network analysis is a subject explored in various fields (computer science, mathematics, physics, biology, social sciences and educational sciences …). At his point in reading, I am wondering whether this definition will become relevant later on. | - As stated just before (comments 15 & 16 in Table 2), in the revised manuscript, we no longer talk about ontology. - The four criteria (soundness, completeness, lucidity and laconicity), all discussed by Guizzardi (2005 & 2007) (e.g., page 22 in his 2007 paper), are used here in order to verify our taxonomy (rows 206-208 and 246-247)and to give recommendations to improve our theoretical classification (see section **Discussion**: rows 771-789 and section **Conclusions** : rows 957-959) - Figure 1 (page 8 of the revised manuscript) was added to explain and represent the four criteria better. |
| 18 | 5 | What is a student network? You have not introduced this term yet. | Definition given in rows 140-142. |
| 19 | 6 | Consider including:   1. Bruun, J., & Brewe, E. (2013). Talking and learning physics: Predicting future grades from network measures and Force Concept Inventory pretest scores. Physical Review Special Topics-Physics Education Research, 9(2), 020109.   Particularly, Figure 3 in the might be interesting as a miniscule precursor idea to your work.   2. Traxler, A., Gavrin, A., & Lindell, R. (2018). Networks identify productive forum discussions. Physical Review Physics Education Research, 14(2), 020107. | 1. Already done when working on the recommendations in Table 1: referenced in rows 161, 184, 391, 392, 919.   For Figure 3 in Bruun & Brewe (2013): See comment number 14 and actions taken in Table 1 for the discussions about the nature of the investigated ties.   2. Added (row 163) |
| 20 | 6 | Hopefully, performances and achievement are related to learning. Perhaps, "other aspects of learning". | Suggestion integrated (row 164) |
| 21 | 6 | ‘*The studies dedicated to student networks mostly conceptualized centrality as (1) the simplest measure of centrality - i.e., the degree centrality, (2) the closeness centrality*  *and/or (3) the betweenness index*’: 🡺 Perhaps a separation between newer work and older work? | Already done when working on the recommendations in table 1 : see Appendix 1 : ‘*Studies on networks and centrality measures*’ and rows 167-176 |
| 22 | 6 | ‘*For instance, in regards with student performance, while the degree and closeness centralities seemed to have a positive effect on achievement, the impact of betweenness centrality appeared to be less clear*’ 🡺 As a reader, I am now curious as to which one's showed what and why that would be. Perhaps a kind of a table indicating what links represent, what nodes represent (which kinds of students), what the centrality measures, what the non-network measure was, and what the outcome was. This would be really useful. | We added Appendix 2, which shows (non exhaustive) instances of college student networks' studies about the links between centrality and academic achievement. (mentioned in rows 182-183 in the revised manuscript) |
| 23 | 7 | ‘*a node has high scores on some centrality measures, but average or low scores on other indicators of centrality*’ 🡺 are these non-network indicators? If so, give examples. If not, use "centrality measure". | As suggested, the word ‘*indicator*’ was replaced by ‘*measure*’ (row 190) |
| 24 | 7 | ‘Finally, as stated earlier, many other metrics than degree, closeness and betweenness were developed in SNA in order to assess a node’s centrality within a graph’ 🡺 See eg. Costas et al (2007) | Reference added to the other references (row 70) |
| 25 | 7 | ‘*One additional objective of this paper is to highlight which centrality measures might be the most informative for student networks. This question has received little attention in student networks research, and the literature on the topic mainly concerns other types of networks than student graphs (e.g., biological networks, terrorist cells, customer networks)*’. 🡺 What have these people found? | This is not developed in the paper since we focus on friendship student networks, and in order to keep the paper to a reasonable length. Appendix 1 shows, however, the centrality indices investigated in those various fields. |
| 26 | 7 | ‘*This work aims at selecting the best suited centrality measures for representing a student network*’ 🡺 You will never be able to argue for this using any one data set. The best you can hope for is to contribute to the discussion by making the argument for your particular network. | This issue has already been treated in comments and actions taken in Table 1:   - The scope of the paper was reduced to one single friendship student network throughout the entire manuscript. - Section ‘**Discussion’**:   We emphasized that future studies could replicate our methodology on other (types of) student networks in order to validate the best centrality measures when we investigate such graphs (rows 901-904; 910-914) |
| 27, 28 & 29 | 7 & 8 | 1. *Section 1.5*. : ‘*The application*   *of the methodology on a real student network allows to*’ : points 1 to 4. 🡺 So, I do not really think that what you have written so far builds up to these criteria. I cannot see that you have shown (1)-(4) to be necessary. However, I think that you could with a few sentences do that in what comes before this.   2. ‘*centrality measures that are the most representative and significant for representing a student network*’ 🡺 a particular student network | 1. The introduction has been rewritten and those issues have already been treated in comment number 3 and actions taken in Table 1. 2. The scope of the paper was reduced to one particular friendship student network throughout the entire manuscript. |
| 30 | 8 | ‘*PCA is applied on the computed centrality measures*’ 🡺 What is your methodological reasoning behind using PCA? How will that connect your data to your theoretical proposition? | This has already been treated in comment number 7 and actions taken in Table 1. |
| 31 | 9 | ‘*We obtained a list of suitable centralities based on our student network’s topology by using the package CINNA (Ashtiani & Jafari, 2017)*’ 🡺 Why did you choose this package? How do you know that they are suitable? What are the warrants given by Ashtiani & Jafari that they are particularly suitable for student networks? | - The package lets one compute a great variety of centrality indices, and is able to treat directed and un-weighted networks (i.e., which is the case for our graph): rows 274-276. - Moreover, the issue was discussed in comment number 15 and actions taken in Table 1. |
| 32 | 9 | ‘*Who are the nodes, what is the network’s type? (i.e., in our case, a directed graph of students)*’ 🡺 But that does not tell us much about the type, in my opinion. What do links represent should be considered as well. | Already treated and replaced by *‘i.e., in our case, a directed friendship student network - see the data section for the details*’: rows 283-284. |
| 33 | 9 | ‘*Indices considered as irrelevant in regard to our network and to research questions related to the network were not selected for further analyses*’ 🡺 how did you do this consideration? | We added an example to show why some centrality indices were eliminated (rows 288-300). |
| 34 & 35 | 9 | 1. ‘*For the remaining indices: we analyzed if there were any measures whose formula was highly similar (i.e., that differ only by very few parameters)*’. 🡺 Formulae that look similar may behave differently in some cases, so shouldn't this be an empirical question? Or at least rely on a mathematical analysis. 2. ‘*Centrality measures that figured in very few documents on Google Scholar were not selected for further analyses*’. 🡺 This choice may be problematic, since you will then contribute to circular reasoning. If people follow your lead, then new measures may never get the chance. | We added those concerns by using your proposals in the revised text in the **Discussion** section that concerns the paper limitations (rows 826 – 838). |
| 36 | 10 | ‘*Based on examples of taxonomies from Song & al. (2015), Lü & al. (2016); Ghazzali & Ouellet (2017) and Ashtiani & al. (2018), who each worked on different sets of centrality measures, we propose five theoretical categories to classify the eighteen centrality measures 273 (see table 1)*’ 🡺 It would be helpful if you summarized these in a table. | That would be very difficult to do, since they each worked on different sets of centrality measures. The summary might therefore not be very clear. Moreover, our taxonomy is - in part - based on the proposals of the classification by those authors. |
| 37 | 11 | ‘*… the first category - i.e., distance-based - assesses the proximity of a node with the other members of the graph. The second category - i.e., geodesic path-based - is related to the geodesic paths in which nodes are located. The third category is based on the connectivity or degrees, i.e., on the number of direct connections a node possesses. Finally, the fourth and five categories take the neighborhood of a node into account, the former the neighbors’ prestige and the later the topology of the members adjacent to the node under consideration*’.  🡺IN my view, this is a very weak theoretical analysis. What is the meaning of these categories in connection to the variety of student networks that exist? How do you think they are related to outcome variables and why? | - Links with (friendship) student networks and outcome variables: this has already been treated in comment number 6 and actions taken in Table 1. - The Appendix 4 also gives the theoretical analysis for the eighteen centrality measures. |
| 38 & 39 | 12 | 1. ‘*PCA enables for identifying the factor on which a variable* ***saturates*** *the most*’ 🡺 Comment: Interesting word - do you mean where the variable has the highest loading? 2. ‘*We performed PCA to determine the most representative centrality measures*’ 🡺 Comment: How did you use PCA to identify the most representative measures? | This has already been treated in comment number 7 and actions taken in table 1. |
| 40 & 41 | 12 | 1. ‘*Students … were interrogated about their friendship ties at university*’. 🡺 Interrogated? That sounds harsh. Perhaps rephrase this. Also, I would suggest that you somewhere present the survey as given to the students. Literature suggests that the generator question is a crucial part of how to understand our networks. See eg. Scott & Carrington (2011). 2. ‘*In the survey, the student’ friends*   *were described as the ‘persons with whom students spend personal time, with whom they interact on a regular basis (in face to face, by phone or on online social medias), that they see outside classes, that they trust, and/or with whom they share their personal issues*’ 🡺 Ok, so *now* I discover that this is a purely social network. In principle, it has nothing to do with academic performance. This is the nature of the network. From a theoretical standpoint, you should consider what kinds of information may flow in this network, and what a reasonable outcome variable might be (persistence? grades? etc.) | - ‘*They were interrogated*’ replaced by ‘*They were asked*’ (row 509) - As stated before, the scope of the paper was reduced to friendship student networks throughout the entire manuscript - Impact of the nature of the investigated ties on educational outcomes such as performance: This has been treated in comment number 14 and actions taken in Table 1. |
| 42 | 13 | ‘*This imputation allowed for computing centrality measures for each of the 870 students belonging to the augmented network*’ 🡺 I like this idea and would like to know more about how it works and how it has been empirically verified. A couple of sentences about that would be nice. | This has already been treated in comment number 9 and actions taken in Table 1. |
| 43 | 15 | I think the English/American convention is to use . and not , -- so 1.00 and not 1,00. | This has already been treated in comment number 20 and actions taken in Table 1. |
| 44 | 16 | I would prefer you to show a table with the results of the PCA. | See tables 2, 3, 4 and 5 (pages 22- 25 of the revised manuscript) |
| 45 & 47 | 16 | Adding references for the three conditions related to relevance of the PCA | - Condition 1: row 539 - Condition 2: row 560 - Condition 3: row 562 |
| 46 | 16 | ‘*Second, the Bartlett's test verifies whether highly correlated variables might be correlated to the same latent factor(s) (Malhotra & al., 2007; Hair & al.,*  *2010). Being significant (p-value = 0.000), the PCA seemed relevant.*’ 🡺 What was significant? what does the p-value pertain to? Maybe leave this out? | To show that the p-value pertains to a chi-square test, we added ‘*(χ² = 20512,07; p-value = 0.000)*’ (rows 560-561) |
| 48 | 16 | ‘*Concerning the quality of a variable’s representation, 50% of the information contained in each variable must be preserved in the factorial plan (Hair & al., 2010), which was the case for all our eighteen centrality measures*’ 🡺 I would need the results of your calculations to verify this. | Table 3 added (page 23) |
| 49 | 16 | What do you mean by inertia? | i.e., variance (added in row 573). |
| 50 | 18 | ‘*Within the taxonomy, four indices are gathered within a first category (i.e., the category number 1 in Table 1), which is built on the criterion of a geodesic distance-based formula and on the criterion of access to information as centrality corollary …*’ 🡺 Since I find the theoretical taxonomy very weakly described and warranted, I find it hard to see that this discussion is interesting. I would much rather have you dive more into what the measures actually measure and what this may mean in a student friendship network. | - The justification and construction of the theoretical taxonomy for student friendship networks was better described and warranted since we justified and explain the choice of the eighteen centrality indices in regard of a student friendship network (see comment number 5&6 and actions already taken in Table 1). - **Results**: Section 5.2. (*The centrality latent dimensions within friendship student networks*) and Section 5.4. ‘*The most representative measures of centrality for friendship student networks*’ treat two of the purposes of the paper (i.e., what the measures actually represent, and what this may mean for a student friendship network). - **Results**: The section 5.3. ‘*The verification of the taxonomy on real data: a friendship student network*’ treats another purpose of the paper. |
| 51 | 19 | ‘A unique dimension (i.e., the dimension number 2) *validates the proximity of indices that were* proposed as belonging to two theoretical categories’ 🡺 what does this mean? | In the revised manuscript: replaced by ‘*A unique dimension, i.e., Dimension 2, which includes and represents three indices, validates the membership in a unique class for those indices that were theoretically proposed as belonging to two theoretical categories, i.e., the third and fourth categories in Table 1)*’ (rows 660-663). |
| 52 | 21 | Table 7: Are all factors represented here? | The table covers the eighteen centrality indices and their average contributions on the factorial plan, not the six latent factors resulting from the PCA. |
| 53 | 22 | ‘*(e.g., between the eccentricity and the closeness centralities, between the geodesic k-path centrality and the betweenness index, between the closeness centrality and the eigenvector prestige score****…****)*’ 🡺 …:??? | The points were deleted (row 722) |
| 54 | 23 | ‘*As far as student networks are concerned, these results suggest that students highlighted as central, because they are cited by many other students having a high degree of Page rank, are also significant through a high number of neighbors that can reach them (i.e., those neighbors being located at maximum k steps towards them)’*. 🡺 This may be true for this network without holding in general. | - ‘*Friendship student networks*’ instead of ‘*student networks*’ (row 747) - ‘*Might also be significant*’ instead of ‘*are also significant*’ (row 749) - In the discussion, we emphasized the need to validate the latent dimensions on other (friendship) student networks: rows 901 – 903) |
| 55 | 23 | ‘*On this basis, we might infer that students having a high Page rank score are geographically close to the student(s) they cite, and belong to the same neighborhood as these students’ closest neighbors.*’ 🡺 How can you infer geographical closeness from this? This would entail knowing where the students actually live in real life? | The geographical concept was related to the graph (not for instance where the students actually live). We specified it in the revised manuscript (row 751). |
| 56 | 23 | ‘*The five proposed categories of the theoretical classification are for the most part validated, since they match the latent dimensions highlighted by the PCA*’ 🡺 My problem with the theoretical taxonomy aside, is "for the most part" enough? What was your success rate and is it acceptable? | - We used the four criteria (i.e., sound, complete, laconic and lucid) to explain to what extent our proposed theoretical taxonomy differs from the real dimensions that were highlighted by using PCA on real data, and how our theoretical classification might be improved. (rows 771-789) - We added the comparison of our verified theoretical classification with categories of centrality indices suggested by other authors in order to support its acceptability (rows 791-812). |
| 57 | 25 | If a variable is not well represented in the factorial plan, its proximity with other(s) variable(s) on the factorial plan may be misinterpreted (i.e., proximity may be assimilated as correlation whereas it is not the case in reality). 🡺 what do you mean with "proximity" and "assimilated as correlation" | The proximity between variables within the factorial plan means that they are correlated and related to the same latent construct. In the revised manuscript, we replaced the passage by ‘*If a variable is not well represented in the factorial plan, its correlation with another variable or other variables may be misinterpreted, i.e., variables might be considered close whereas that is not the case)*’ (rows 841-844) |
| 58 | 25 | Since the respondent only approach  might produce more biased estimates than the imputation approach (e.g., Huisman, 2009; Wang & al.,2016; Gile & Handcock, 2017) we chose to impute ties for the non respondents, by means of ERGMs, which give better performances than simpler imputation procedures (Huisman & al., 2018). 🡺 Have you tried both to see if it makes a difference to your categories? | Yes, see comment number 16 and actions taken in table 1. |
| 59 | 26 | ‘*Other measures than those that were chosen in this paper should be tested.*’ 🡺 I think you should do this in this paper. | See comment number 15 in table 1. |
| 60 | 26 | ‘*Centrality measures considered as appropriate for a given network may not be able to correctly identify central nodes within other graph typologies*’ 🡺 But, how do you establish, what is correct here? | - By having computed - in Section 5.4. - the most representative measures of centrality for our friendship student networks. - And by insisting on the need to replicate the study on other friendship networks in the discussion section (rows 901 – 904). |
| 61 | 26 | We argue that the choice and identification of the best centrality candidates should be the first step when investigating networks and when identifying its key players. 🡺 But, I have not seen the warrants for this argument. Why is this better than just testing all known centrality measures? | Comment 15 in Table 1: ‘*Jalili et al. (2015) identified 113 centrality measures; it would be very difficult, even impossible, to include all those measures in only one study* 🡺 Added in the revised manuscript (rows 927-929). |
| 62 | 26 | ‘*Results also show that*’ 🡺 suggest that | Replaced in the revised manuscript (row 947) |

We thank you again and remain entirely at your disposal for any other comment(s) or question(s)!

1. The reference is not mentioned here in order to ensure the double-blind review process.
