# Supplementary material for: A methodology and theoretical taxonomy for centrality measures: What are the best centrality indicators for student networks?
Source: PLoS One. 2020 Dec 30;15(12):e0244377. doi: 10.1371/journal.pone.0244377 (PMC7773201; doi:10.1371/journal.pone.0244377)
Supplement: Attachment — Submitted filename: Response to Reviewers.docx [file pone.0244377.s011.docx]

Response to Reviewers

We would like to warmly thank you again for the very constructive comments that were made and enabled us to improve the paper. We remain entirely at your disposal for any other comment(s) or question(s)!

| N° | Comment(s) | From | Action(s) |
| --- | --- | --- | --- |
| 1 | Maybe it would be beneficial for the credibility of this paper to highlight the exploratory nature of this study in the introduction. It has to be pointed out that most of the issues mentioned above cannot be resolved in the present form. With proper framing, this should not be an issue though. | Reviewer 1 | Rows 138-140: ‘*This research is exploratory and constitutes a first step to the development and applying of methodologies that compare and validate centrality measures which highlight central nodes within networks*’.  Rows 1089-1091: ‘*exploratory research methodology that we propose may constitute a first step when investigating some network(s) of interest*’. |
| 2 | Do mind some language errors such as “all types of network” in line 171 and “the second step consists in selecting” in line 291, among others. | Reviewer 1 | Corrections underlined in green in the revised manuscript |
| 3 | **Introduction**  ‘In essence, I believe that this section should convince the reader that the problem of finding out to what extent centrality measure the same "aspect of reality", is worthwhile’.   1) Line 55: "First, it is crucial to classify..." This sentence is a postulate that appears without backing. Backing could come in the form of a reference, a further explanation or (preferably) both. One approach could be to point to other papers which have found correlations between centrality indices but have not treated these correlations any further. 2) Line 57: The manuscript claims that closeness and betweenness centralities are most used. In fact, they have data to prove that, but the reader does not know this at this point. I suggest leaving out the specific mentioning of particular centrality measures. 3) Line 58: "Alternative measures could be considered..." Yes, but why would we want that? Explain this to the reader in a short phrase. 4) Line 59: "The appropriateness ... should also be taken into account" Why? What would we gain from this? 5) Line 72: I do not see the logical imperative from the scarcity of reviews on centrality measures to thorough *definitions*. Rather, I would like to know, what centrality measures might mean in the context of student friendship networks; in the network of interest. In my opinion, the literature is abundant with rigorous definitions (e.g. Wasserman & Faust 1994, Costa et al 2007). 6) Line 85: "rare taxonomies of centrality that existed..." Which - provide reference? 7) Line 87: "it is crucial to verify ..." Why? Why will we potentially achieve by verifying these things? Better predictions? Better predictive models? Other things? 8) Line 93: Only few centrality measures have been used... You can actually show this with your literature review. Perhaps rewrite this to say: "...*it seems* that only a few have been used." 9) Line 96: How do we know that central actors have been identified correctly? 10) Also, either in the end of the introduction or in the end of Section 2, as a reader, I would very much appreciate clearly stated research questions in the form of:   "Research Question 1: Which theoretical and empirical taxonomies can be extracted using our PCA-based methodology for centrality measures?" <- my attempt... | Jesper Bruun | 1) Rows 55-58: ‘*Many studies (e.g., Rothenberg & al., 1995; Lee, 2006; Valente & al., 2008; Bruun & Brewe, 2013; Ghaffar & Hurley, 2020) found correlations between several centrality indices within different type of networks. However, those researches did not deepened their results, i.e., explained their results in detail and /or classified the centrality indices based on the correlations that were found*’. 2) Rows 61: The specific closeness and betweenness centralities were deleted. 3) Rows 62-64: ‘*since each centrality measure that has been developed in SNA differs in its meaning of centrality, using several measures in networks studies could bring more information about nodes centrality*’. 4) Rows 64-67 : ‘*Moreover, the appropriateness of the centrality measures should also be taken into account when studying networks, since each graph is different as far as its nature is concerned (e.g., its type: social, biological, financial, etc.; the direction of its edges: directed versus undirected)*’. 5) Rows 78-84: ‘*Furthermore, it is crucial to understand centrality measures in light of the network(s) of interest, i.e., in the context of the studied graph(s). Glossaries that thoroughly describe and define centrality measures within networks can serve this purpose. One of the objectives of this paper is to explain in detail a number of centrality measures found in the literature, that is, to give thorough definitions about those centrality indices, together with their formulas, in order to understand those indices in light of student networks, i.e., of our graph of interest.*’. + Rows 1064-1065 6) Added in rows 90-91 7) Rows 97-100: ‘*However, in order to be sure to classify within same categories measures of centrality whose meaning is similar, and to separate within different categories centrality indices that are different in nature, it is crucial to verify the validity, accuracy, and appropriateness of the proposed classifications of centrality indices by means of criteria that are relevant for taxonomies*’. 8) Rows 105-106: ‘*Third, as stated before, many centrality measures exist in SNA, but according to our literature review, which is detailed further in the paper, it seems that only a few have been used*’. 9) Reformulated: rows 108-109: ‘*Also, the ways centrality is defined and computed might be relevant for best identifying central actors within graphs*’. 10) Rows 141-153 :   ‘*The four empirical research questions that are investigated in this paper are:*  *Research question 1) Which centrality measures should be chosen among a large set of indices because they seem relevant for friendship student networks?*  *Research question 2) Which theoretical taxonomy might allow classifying those chosen indices?*  *Research question 3) By using PCA on those centrality measures, which are the centrality dimensions that are highlighted, and does the proposed theoretical taxonomy align with those dimensions?*  *Research question 4) Which are the representative centrality indices for friendship student networks?*’ |
| 4 | **Theoretical section**  While Table 1 in Appendix 1 is informative, you could easily convert this into a graphical representation in the paper. Either a bar graph showing the ten most used measures or a word cloud. This is meant as a suggestion, only. | Jesper Bruun | Figure (Fig 1.) added (row 206) |
| 5 | **Section III: A proposed ...**   1) Line 489: For me it is really weird that this is not an equation. In my opinion, the quantity should be called some letter (eg. alpha), because then you can easily reference this quantity later on. 2) Line 490: Please write out the summation for \lambda for clarity. | Jesper Bruun | Changes made (see rows 521-528) |
| 6 | **Section IV: Data**  Here, I really miss an overview of the two networks (the "raw" as well as the imputed). I would like: number of nodes, N, number of links, L, diameter, average shortest path length, a summary of the degree distribution (like the power law exponent if they follow a power law or an average degree). I would also prefer a graphical representation of each network to see how they look different. For example, seeing imputed links would be really good to see. | Jesper Bruun | Rows 569-625: Section 4.2. ‘*Comparison between the respondents’ network and the augmented network: visualization and structural properties of the two graphs*’.   - Fig 5. Representation of the original network and of the augmented network - Table 2. Structural properties of the respondents’ network and of the augmented network. - Fig 6. Distribution of node degree for the two networks |
| 7 | **Section V: Results**   1) Since the authors have now made the calculations for the non-imputed network, I think they should include them throughout the paper (eg. in Table 6). This would increase the reader's understanding of the methodology (here, the impact of imputing links) 2) In Table 6, the authors could consider labeling Latent dimensions with "latent dimensions ("reality")" and "theoretical constructs" to make the connection to Figure 1 clear. 3) In Table 7, if these are averages, what are the uncertainties? | Jesper Bruun | 1) Done: section ‘*5.4. Generalization and summary of the results*’ : rows 753-808 2) Done (🡺 now table 7) 3) These averages are computed by means of the Formula 1 (rows 520-525; formula that is briefly reminded in the section 5.4.). Therefore, we do not have the uncertainties. |
| 8 | **Section VI: Discussion**   1) Line 768: "are for the most part validated" - I do not think this wording is appropriate. I would start this section more "matter of factly": "We now turn to the question of whether the theoretical model could be validated." (or something like that). Then sum up which part of Figure 1 are validated and which are not (and how), as you do on the next page. 2) Lines 778-780: I am uncertain why the authors have placed the number 2 in the bottom right of Table 6, since they say here that a new category has to be proposed to achieve completeness. To make the point that the theoretical model is not complete that slot should be empty. 3) Lines 782-784: If you make 3+4 into one theoretical construct, would that not also point to two different empirical ("real") objects? 4) Also, if the authors keep 2 in Table 6 then there’s another theoretical concept which points to two empirical objects. 5) Lines 786-790: Here I would add that page rank also needs to be reinterpreted for the mapping to be laconic. 6) Also, in an appendix, it would be nice to have the distributions of the data (each of the 18 centrality measures). These can be easily computed in R. if their distributions differ a lot from normal distributions, this may influence the reader's view of the correlations. | Jesper Bruun | 1) Done : rows 891-908 2) Table 7 confronts the theoretical taxonomy (that we proposed originally) with what we observed in reality (i.e., the six dimensions from the PCA). Then, in the discussion section, we propose to improve our original taxonomy, based on the differences that were highlighted between this original taxonomy and reality. For more clarity, table 7 was reworked, with the proposed theoretical categories in the first column instead of the last column of the table.   Row 787-788: ‘*Table 7 compares the centrality dimensions that came out of the PCA (for both networks) with the theoretical categories proposed within the taxonomy, and summarizes the above findings*’.   3) No, because there is a unique dimension (i.e., a unique reality) for the objects that were originally proposed as belonging to two different theoretical constructs. One theoretical construct is therefore sufficient in order to point to only one real object, i.e., only one dimension of centrality. For more clarity, we replaced ‘*3 & 4*’ in table 7 by ‘*3 or 4*’. 4) Table 7 shows that we originally proposed the two bottlenecks scores as part of the second theoretical category, but that the two scores were part of another dimension in the reality.  - see rows 919-921 : ‘*In order to be complete (i.e., to cover each aspect of the centrality notion within a friendship student network), an additional category within the theoretical taxonomy should be proposed for the bottleneck centrality*’  5) Added : rows 931-934 : ‘*The page rank score would also needs to be reinterpreted for the taxonomy to be laconic, since this centrality measure was proposed as belonging to two theoretical categories, whilst being part of only one dimension in reality*’. 6) Discussed in rows 1001-1016, and Appendix 7 (added). |
| 9 | **Section VII: Conclusion**  Once the authors have the made research questions, they should also explicitly state what the answers are to the research questions here. | Jesper Bruun | Done : rows 1069-1071, 1080, 1081, 1085 |
