# Supplementary material for: A methodology and theoretical taxonomy for centrality measures: What are the best centrality indicators for student networks?
Source: PLoS One. 2020 Dec 30;15(12):e0244377. doi: 10.1371/journal.pone.0244377 (PMC7773201; doi:10.1371/journal.pone.0244377)
Supplement: Attachment — Submitted filename: Response to Reviewers.docx [file pone.0244377.s012.docx]

Response to Reviewers

Dear Sir Ward, dear sir Brunn,

Thank you again so much for the very constructive comments that you made and enabled us to improve the paper! We remain entirely at your disposal for any other comment(s) or question(s)!

| N° | Comment(s) | From | Action(s) |
| --- | --- | --- | --- |
| 1 | With regard to language, the changes that have been made in green are not always correct:  - The fixed collocation with '*consist*' is '*consist of*'. An example would be: '*the second step consists of selecting*'.  - '*type of information*' should be *'types of information'*, '*other type* *of graphs*' should be '*other graph types*', '*other type of networks'* should be '*other network types'*, etc. | Peeters Ward | In green within the revised manuscript |
